# A Blockchain-Based Authentication and Authorization Scheme for Distributed Mobile Cloud Computing Services

**DOI:** 10.3390/s23031264

**Published:** 2023-01-22

**Authors:** Linsheng Yu, Mingxing He, Hongbin Liang, Ling Xiong, Yang Liu

**Affiliations:** 1 School of Computer and Software Engineering, Xihua University, Chengdu 610039, China; 2 School of Transportation and Logistics, Southwest Jiaotong University, Chengdu 611756, China

**Keywords:** authentication, authorization, access control, mobile cloud computing, blockchain

## Abstract

Authentication and authorization constitute the essential security component, access control, for preventing unauthorized access to cloud services in mobile cloud computing (MCC) environments. Traditional centralized access control models relying on third party trust face a critical challenge due to a high trust cost and single point of failure. Blockchain can achieve the distributed trust for access control designs in a mutual untrustworthy scenario, but it also leads to expensive storage overhead. Considering the above issues, this work constructed an authentication and authorization scheme based on blockchain that can provide a dynamic update of access permissions by utilizing the smart contract. Compared with the conventional authentication scheme, the proposed scheme integrates an extra authorization function without additional computation and communication costs in the authentication phase. To improve the storage efficiency and system scalability, only one transaction is required to be stored in blockchain to record a user’s access privileges on different service providers (SPs). In addition, mobile users in the proposed scheme are able to register with an arbitrary SP once and then utilize the same credential to access different SPs with different access levels. The security analysis indicates that the proposed scheme is secure under the random oracle model. The performance analysis clearly shows that the proposed scheme possesses superior computation and communication efficiencies and requires a low blockchain storage capacity for accomplishing user registration and updates.

## 1. Introduction

In recent years, with the increase in the number of smart mobile devices and the popularization of cloud computing technologies, mobile cloud computing (MCC) as a novel computing paradigm has received extensive attention and significant development [1]. A mobile user can use resource-limited mobile devices to subscribe to and access the remote cloud computing services from different types of service providers (SPs), e.g., Netflix, Apple Music, and Amazon cloud drive [2]. The distributed locations of SPs enable subscribers to access the resources efficiently and conveniently [3]. However, the MCC environment possesses the characteristics of openness and complexity since it is dependent on insecure wireless technology [4]. Hence, to protect the interests of users and SPs, it is essential to provide vital security assurances, especially authentication and authorization [5]. In addition, the quantum-safe authentication protocol for mobile devices has recently been proposed to resist quantum computer attacks [6].

**Figure 1:**
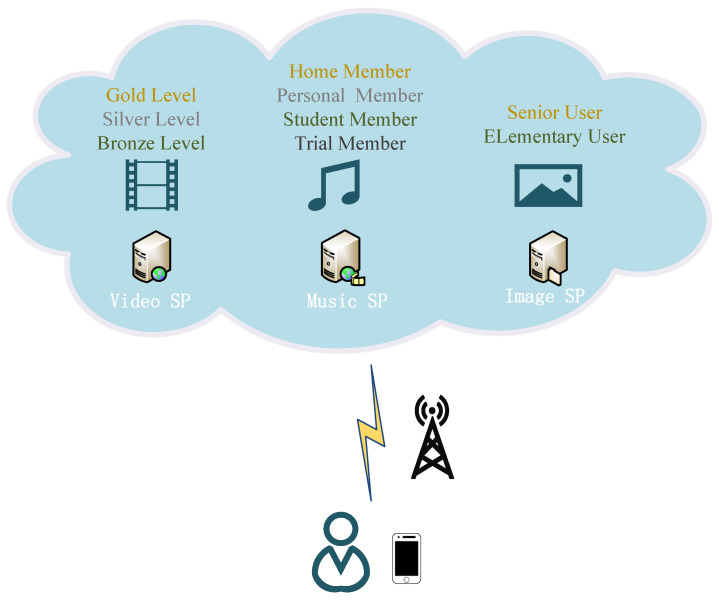
The architecture of MCC hierarchical services system.

The openness of wireless communication causes the data transmitted between mobile users and SPs to be easy to maliciously intercept and modify [7]. The attacker is likely to impersonate a legitimate mobile user or SP to steal benefits. Thus, authentication has been widely applied to assist users and SPs to identify each other [8,9,10,11,12]. In addition, a kind of cloud service is usually divided into several levels, e.g., ‘Bronze’, ‘Silver’, and ‘Gold’, to satisfy the different subscription demands of users [13,14]. Authorization is essential for SPs to determine the access privilege levels of users as they use a hierarchical cloud service [15]. For example, as shown in Figure 1, a mobile user subscribes to a ‘Silver’ level of cloud video service from a video SP. When this user accesses the video SP to use the service, the video SP verifies the user’s identity and provides a ‘Silver’-level service based on the user’s access privilege. Therefore, authentication and authorization compose an indispensable security component, i.e., access control, preventing malicious adversaries from accessing resources and low-level users from accessing higher-level services [16].

Mobile users can subscribe to new types of services or update their service levels anytime and anywhere in the distributed MCC environment. However, when a user intends to subscribe to multiple different types of hierarchical services, the user usually needs to register on different SPs and maintain corresponding credentials inconveniently, e.g., accounts, private keys, or passwords. Instead, it is more convenient for users to use only one certificate to access hierarchical services from different SPs. This security requirement is called single registration [17]. The obstacle to single registration is that there is generally no trust relationship among isolated SPs to manage user registration information jointly [18].

In addition to introducing a trusted third party, blockchain is an effective technology for establishing a trust relationship in the distributed environment [19]. The distributed trust is assured by the immutability of the blockchain, as well as automatic and transparent smart contracts [20]. Therefore, blockchain is widely used in access control schemes for distributed scenarios, e.g., information-centric networking (ICN) [21] and Internet of Things (IoT) [22]. Moreover, there are usually two types of access control designs. One is where a subject (requester) sends a transaction to trigger the smart contract deploying the access policy in order to access resources [23,24,25]. This method is not effective enough for the MCC scenarios since the blockchain needs to store the enormous number of resource-access transactions submitted by mobile users. Another promising approach is where a subject’s access privilege is stored in the transaction for the object (resource) owner to make authorization decisions when the subject accesses resources [26,27,28,29]. This method is more suitable for the MCC scenarios, since it avoids the mobile user submitting frequent access requests to the blockchain.

### 1.1. Motivation and Contributions

Single registration has been widely considered in multi-server authentication schemes. Some centralized multi-server authentication and authorization schemes, using a registration center (RC), also inherit this advantage [9,15]. However, all users and SPs need to fully trust the RC, leading to expensive trust costs and single-point failure [12,30,31]. In the distributed MCC hierarchical service scenarios, we believe that achieving single registration without a trusted third party has the following characteristic: a mobile user just needs to register on an arbitrarily selected SP once and can subscribe to hierarchical services on other SPs concurrently; then, the mobile user can use one credential to access multiple SPs to use services with different levels. In order to make this idea work, the following three security issues need to be considered: (1) when a mobile user registers, identity forgery should be prevented; (2) the mobile user’s subscription request should be checked in a trusted way, and not by the user’s selected registration SP; (3) subscription fees paid by a mobile user belonging to other SPs cannot be embezzled by the selected registration SP. This paper uses the non-interactive zero-knowledge (NIZK) proof of knowledge and blockchain technology to solve the above issues: (1) the registration SP verifies NIZK proof of knowledge to confirm the validity of user identities; (2) the smart contract checks the user’s subscription request and decides whether to complete the registration; (3) other SPs ask for their deserved fees from the selected registration SP based on the tamper-proof distributed ledger. In these ways, all SPs trust that a mobile user registered on an arbitrary SP is also their legitimate subscription user, enabling the single registration.

Using blockchain to design an authentication and authorization scheme possesses the advantages of a low trust cost and single-point-of-failure resistance. However, it is also faces some issues, especially storage overhead [32,33]. In other distributed scenarios, generally, an object owner generates a transaction to record a subject’s access privilege [26,27,28,29]. Afterwards, the object or owner make access control decisions by reading the authorization transaction. However, when a subject requests access privileges from multiple object owners, it means that multiple transactions are required to be stored in blockchain, since each transaction only loads one authorization relationship between a subject and an object owner. A subject’s multiple authorization transactions aggravate the storage burden of the blockchain. Especially in the MCC scenarios, it is hard to ignore the blockchain storage pressure caused by a numerous number of mobile users and SPs. Therefore, the proposed scheme consolidates multiple authorization relationships between a mobile user (subject) and multiple SPs (object owners) into one transaction. In this way, when a user registers, even if there is a large number of SPs, the different access privileges of a mobile user only need to be stored in one transaction. This reduces the storage overhead of blockchain and improves the system scalability.

In practical applications, user access privileges updates should be considered for user convenience. This enables mobile users to subscribe to new levels of hierarchical services at any time. User access privileges updates, in our scheme, can follow the feature of the registration phase: a mobile user can update access privilege levels on multiple SPs at once, and all updated access privileges only need to be stored in one transaction. However, it requires two additional considerations. First, mobile users should not pay repeatedly when updating their unexpired access privileges. In other words, mobile users can inherit the service time that has not been used up before. Second, the immutability of the blockchain makes it impossible to delete and modify privileges, causing mobile users to reuse previously expired privileges, i.e., double-spending attack [34]. The proposed scheme uses the smart contract deploying flexible update strategies and the world state to solve the above two issues, respectively [35]. The contributions of this work are mainly summarized as follows:

- This paper proposes a blockchain-based authentication and authorization scheme for distributed mobile cloud services that enables the mobile user to access different SPs with different access levels by using the same identity and credential. In addition, the mobile user just needs to register on an arbitrary SP once to apply for access privileges from different SPs with the participation of the smart contract.
- Each time a mobile user registers or updates, only one transaction needs to be stored in blockchain to record the user’s access privileges on multiple SPs. The detailed theoretical and experimental analysis indicates that the proposed scheme alleviates the storage burdens on the blockchain and provides scalability when the number of SPs and users increases.
- The proposed scheme utilizes the smart contract to achieve efficient and flexible user access privilege updates, and uses the world state to resist the double-spending attack. We implemented the smart contract algorithms for the registration phase and update phase on Hyperledger Fabric.

### 1.2. Organization

The rest of this paper is organized as follows. Section 2 reviews the related works. Section 3 describes the system model and security model. Section 4 explains the system building block of the blockchain and the transaction structure. In Section 5, we present the details of our proposed scheme. We perform the smart contract experiment in Section 6. Section 7 demonstrates the security analysis of our proposed scheme. The performance analysis is introduced in Section 8. Section 9 discusses our work in depth. And Section 10 summarizes our work and prospects for future work.

The abbreviations and symbols used in this paper are defined in Table 1.

## 2. Related Work

This section will present the situation of the multi-server authentication technology for the MCC environment and blockchain-based access control schemes, respectively.

### 2.1. Multi-Server Authentication Schemes for MCC Environment

In 2015, the multi-server authentication scheme designed for the MCC environment was first introduced in Tsai et al.’s paper [17]. However, [3,8] indicated that this scheme [17] is vulnerable to server impersonation attack. Their schemes avoid the faults existing in Tsai-Lo’s scheme and achieves lower computational overhead and communication costs. In 2021, ref. [36] presented an efficient dynamic reciprocal authentication scheme using a one-time password to achieve multi-factor authentication, which is good at preventing the social engineering attack and replay attack. Ref. [4] not only considers scalability but also avoids using computation-intensive pairing operations on resource-constrained mobile devices. New users or servers are free to join the system, and there are no expensive pairing operations in their scheme to enhance efficiency.

Recently, some designs that combine authentication with authorization have been proposed for the MCC scenario. Ref. [9] combined attribute-based encryption (ABE) and multi-server authentication for MCC healthcare applications. A user with the corresponding access privileges is able to authenticate with the server by decrypting parameters encrypted with ABE. However, the user side needs to perform costly pairing operations of ABE. Ref. [15] designed an authentication and authorization scheme for a hierarchical cloud service. In the authentication phase, SPs determine users’ access privileges in order to reject unauthorized access or authenticate with users. In addition, mobile users are allowed to subscribe to new levels of cloud services on RC at any time.

All above-mentioned schemes suffer from the weakness of single-point failure due to the use of trusted third parties. Some blockchain-based authentication schemes do not require a trusted third party. Ref. [11] proposed a blockchain-based authentication scheme for multi-server architectures. Their scheme allows users to select any server to upload the registration certificate to blockchain for the single registration. Ref. [12] presented a blockchain-based authentication scheme for the MCC environment. This scheme is more efficient in computation but has a higher communication cost than Xiong et al.’s scheme [11]. However, neither of the decentralized designs consider the extra authorization property for hierarchical services by using smart contracts.

More recently, ref. [13] designed an excellent distributed access control framework for pervasive edge computing (PEC) services. Their scheme combines the advantages of decentralization, authentication, and authorization. The PEC server determines the level of service that the user can access according to the plaintext authorization token forwarded by the base station. They adopted an innovative method that utilizes multi-authority ABE to implement decentralized asynchronous authentication. However, their scheme is deficient in user anonymity and privilege updates.

### 2.2. Blockchain-Based Access Control Schemes

This section mainly reviews the blockchain-based access control schemes utilizing transactions or tokens, which is similar to our scheme. Ref. [26] proposed a blockchain-based user private data protection scheme where the user sends a transaction that records the access permissions of the service to their data. In addition, the service can send an access transaction to trigger a permission check, and then access user data from an off-chain database. In CapChain [27], a device owner sends a transaction that delegates device operational capabilities to a user. When a user accesses a device, the device can query the authorized transaction to determine the user’s permissions. FairAccess [22,37] enables the owner of the device to send a transaction that loads their access control policy in order to grant a subject the access token. Then, the subject meeting the access control condition sends a transaction to prove its permissions, and uses the token to access the device.

In BlendCAC [28], the object owner launches a transaction to save a token that records the subject’s authorized operations for the object. After receiving the access request from a subject, the object fetches the token from the smart contract to make the decision to grant access to the object. Ref. [29] proposed an improved method that divides the token of BlendCAC into multiple tokens, and each token is associated with an operation. This improvement achieves a more flexible capability delegation, but it leads to the need for more transactions. In SBAC [21], a content provider generates an access token based on a content requester’s attribute score, and sends a transaction to securely transfer the token on blockchain. Then, the content requester can access the content after the content provider verifies the validity of the received token from blockchain. Ref. [23] proposed a smart-contract-based access control scheme where an access control contract records the authorization relationship of one subject–object pair. The subject can send a transaction for the verification of its access request, and the access control contract will return the validated result to both the subject and object.

These blockchain-based access control schemes solve the threats in traditional centralized access control schemes, e.g., a central leak, single-point failure, and internal attack. However, when a subject wants to access the resources of many object owners, it is required to make multiple requests to those object owners. This is very inconvenient for the subject. Furthermore, these object owners generate multiple transactions or tokens separately, stored in blockchain, to record the subject’s various access permissions. It also increases the storage overhead of the blockchain. Combined with our discussion in Section 1.1, applying blockchain to implement an authentication and authorization scheme for MCC scenarios should consider convenient single registration and low storage overhead.

## 3. Preliminaries

### 3.1. System Model

**Figure 2:**
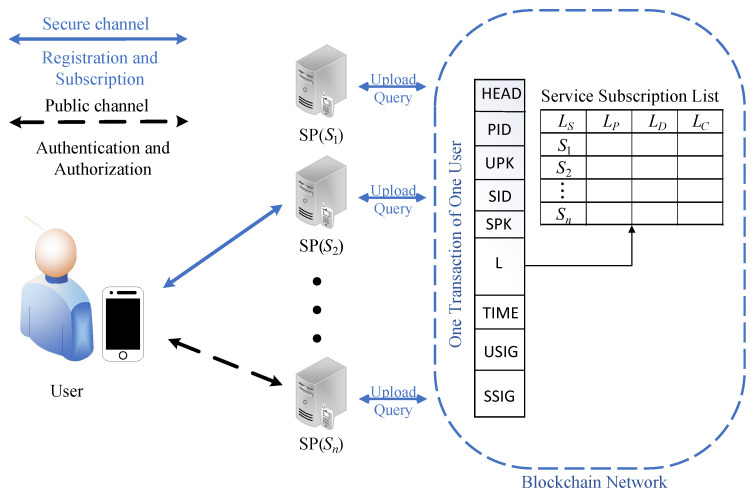
Our system architecture and transaction structure: one transaction records a user’s subscription information on n SPs.

The two types of participants and the blockchain network in the proposed scheme are shown in Figure 2.

1. **Mobile users (**$U_{i}$**)**: Mobile users can use mobile devices to access hierarchical cloud services on all SPs after registering on an arbitrarily selected SP.
2. **Service providers (**$S_{j}$**)**: Each SP in our system needs to be a semi-trusted party. After verifying the mobile users’ identities, privileges, and service periods, $S_{j}$ provides the hierarchical cloud services for them.
3. **Blockchain network**: The proposed scheme adopts an access-restricted but efficient consortium blockchain. SPs constitute the blockchain network as the nodes, and SPs also generate transactions that record users’ identity and subscription information (access privileges, service periods, and service fees paid).

### 3.2. Adversary Model

We assume that there exists a probabilistic polynomial time (PPT) adversary A. The purpose of the adversary A is that A can successfully impersonate an SP or mobile user to authenticate with another mobile user or SP. In addition, A can perform the following feasible attacks:

A can control the public channel between $U_{i}$ and $S_{j}$; that is, the adversary can inject, block, eavesdrop, and tamper with all of the transmitted messages through the public channel.

A can obtain either the mobile device or the password, which are the two authentication factors. Moreover, A can extract the secret information from the mobile device obtained by the adversary. Then, the password space ∣$D_{PW}$∣ can be enumerated by A using offline dictionary attacks.

A is probably a legal but malicious mobile user.

### 3.3. Security Requirements

According to the previous multi-server authentication schemes [3,8,38], our authentication component should satisfy the following security requirements, including mutual authentication, user anonymity and un-traceability, user-friendly password local updates, multi-factor security, resistance to a wrong password login/update attack, and resistance to a reply attack. Furthermore, according to the latest centralized authentication and authorization scheme [15], we believe that a blockchain-based authentication and authorization scheme for the MCC hierarchical service scenario may consider the following security properties.

1. **Single registration**: Mobile users just need to register on an arbitrarily selected SP once to access cloud services provided by multiple SPs.
2. **Hierarchical access control**: In practical applications, the services provided by an SP are usually divided into different access rights. The trial services can be freely accessed by any legitimate user. Other high-level services need to be purchased by mobile users. In the authentication phase, an SP can determine mobile users’ access privileges to authorize users to access the corresponding level of service.
3. **Access within limits of permission**: This guarantees that unauthorized mobile users cannot access the services above their permission level. Moreover, the SP cannot provide services below users’ access privileges.
4. **Efficient and flexible update user access privilege**: Mobile users can update their access privileges at any time. However, expired users can only access the trial service, and even if a mobile user only updates the service privilege on one SP, the user’s access privileges on other SPs must be updated at the same time.

## 4. System Building Blocks

This section will introduce zero-knowledge proof of knowledge, Hyperledger Fabric, the transaction process, the structure of transactions, the service subscription list, and the smart contracts in our scheme.

### 4.1. Zero-Knowledge Proof of Knowledge

In a zero-knowledge proof of knowledge (ZkPoK) protocol [39], a prover can convince a verifier that a statement is true, and the verifier only learns the validity of the statement (without disclosing much else). Following the notation in [40,41], let $ZkPoK\{\left(x\right):y=g^{x}\}$ indicate a ZkPoK protocol that proves the knowledge of $x\in Z_{q}^{*}$ such that $y=g^{x}$. The ZkPoK protocol is able to be transformed into being noninteractive by using the Fiat–Shamir heuristics [42].

### 4.2. Hyperledger Fabric

**Figure 3:**
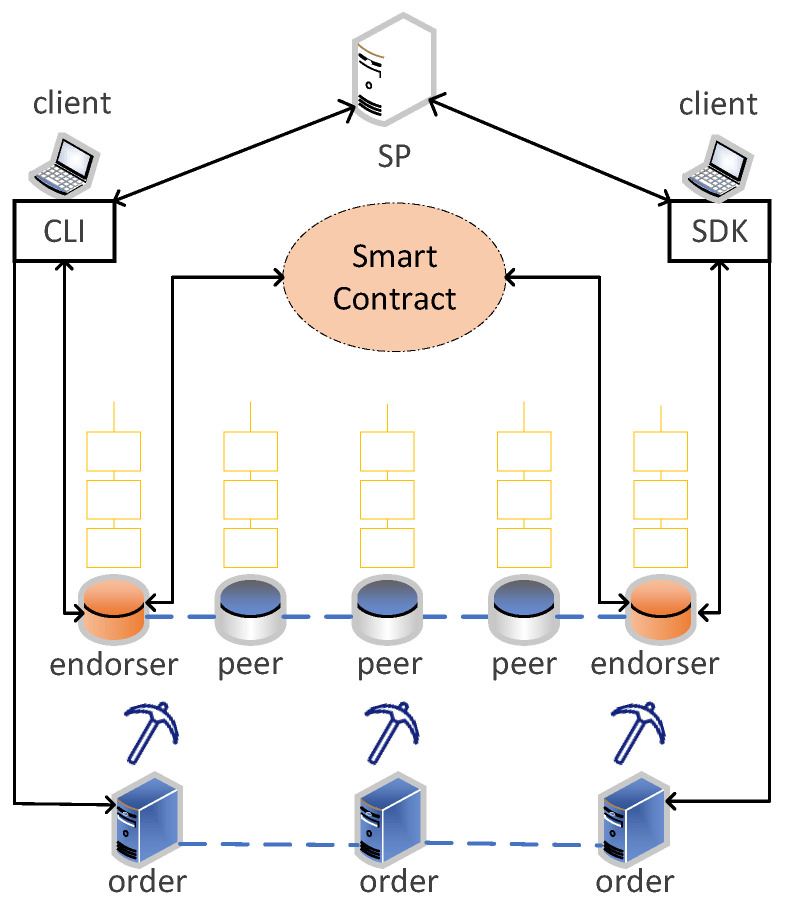
The structure and transaction process of Fabric.

The proposed scheme uses Hyperledger Fabric, or Fabric for short. Fabric focuses on the distributed storage of data in ledgers, which is different from other cryptocurrency blockchain platforms. The smart contract, also called chaincode in Fabric, implements the application logic of the modification, writing, and check, and Fabric deploys a key-value database to store the world state that is the most recent value of transactions. In addition, a modular membership service provider (MSP) used in Fabric makes it more convenient to manage the access rights of organizations. MSP also issues credentials and manages the identities of all nodes in the organizations. The organizations that are joined into the blockchain can own four types of nodes with different functions (peers, clients, endorser, and orders) [35]. The structure of Fabric is shown in Figure 3.

### 4.3. Transaction Process

The transaction process is shown in the Figure 3. The SP invokes CLI or SDK (clients) to upload the transaction to the endorser on the blockchain. Each endorser calls the smart contract to check the correctness of the transaction content, which checks the payment of mobile users in our scheme, and then returns the signed endorsement result to the clients. After collecting enough endorsements, the clients submits the transaction to the orders node. Then, the orders node executes a consensus algorithm, e.g., RAFT [43], to pack the received transactions into blocks, and orders broadcasts blocks to peers nodes that store these blocks. The latest transactions are stored in a key-value database, which can be queried by an SP using the keyword immediately.

### 4.4. Transaction Structure

To reduce the number of transactions, a transaction needs to record a user’s identity information and subscription information on multiple SPs. A flexible transaction structure was used in our scheme, which is similar to the tabular structured ledger [44,45]. In this way, a transaction records the authorization relationships between a user and multiple SPs. The abstract structure and data structure in the transaction are shown in Figure 2 and Algorithm 1 separately.

- **PID:** symbolizes pseudonyms of mobile users.
- **UPK:** symbolizes public keys of mobile users.
- **SID:** symbolizes the identity of an SP who accepts the mobile user’s registration information.
- **SPK:** symbolizes the public key of the acceptance SP.
- **L:** symbolizes the service subscription list, which includes four arrays: $L_{S}$, $L_{P}$, $L_{D}$, and $L_{C}$.
- **TIME:** symbolizes the user’s registration and update time.
- **USIG:** symbolizes the user’s signature value of the registration information sent to the acceptance SP.
- **SSIG:** symbolizes the acceptance SP’s the signature value of the information sent to the blockchain.

**Algorithm 1** Init_Ledger()
1:**Struct** User public {2:      $PID_{i}$             JSON:**PID**;3:      $PK_{ui}$              JSON:**UPK**;4:      $ID_{sj}$              JSON:**SID**;5:      $PK_{sj}$              JSON:**SPK**;6:      $L_{S}$                JSON:**CSP**;7:      $L_{P}$                JSON:**Privilege**;8:      $L_{D}$                JSON:**Day**;9:      $L_{C}$                JSON:**Cost**;10:      *T*                 JSON:**TIME**;11:      $S_{ui}$                JSON:**USIG**;12:      $S_{sj}$                JSON:**SSIG**;13:}%The data structure in the transaction.14:var Init_U User = Null;15:Storing the initialization transaction Init_U in the blockchain;16:**return**Null;


### 4.5. Service Subscription List

In our proposed scheme, $L=[L_{S},L_{P},L_{D},L_{C}]$ is a service subscription list that is used to provide hierarchical access control and subscription during the authentication, registration, and update phase. The detailed structure of *L* is illustrated in Table 2.

The entry $S_{j},Lev_{Sj},Day_{Sj},C_{Sj}$ in Table 2 is explained as follows. $U_{i}$ subscribes to a service with the level of $Lev_{Sj}$ on $S_{j}$ for $Day_{Sj}$ days used, and costs the service fee $C_{Sj}$.

Assume that there are *j* existing SPs and *n*-*j* unjoined SPs. $L_{S}$ = [$S_{1}$,...,$S_{j}$,...,$S_{n}$] is an array of all SPs.

$L_{P}$ is a non-negative integer array that indicates the user’s access privileges on all SPs. In $L_{P}$, the access privileges published on the website are mapped onto non-negative integers. For instance, the levels [0,1,2,...] represent the access privileges {trial-user,elementary-user,intermediate-user,...} separately. In addition, $Lev_{Sj}$ represents the access privilege of the user $U_{i}$ on $S_{j}$.

$L_{D}$ is a non-negative array that indicates the days where $U_{i}$ intends to use the privilege service on all SPs. Furthermore, $Day_{Sj}$ represents the number of days where the user $U_{i}$ uses the privilege service on $S_{j}$.

$L_{C}$ is a non-negative integer array that indicates service fees for all privilege services paid by $U_{i}$. Moreover, the service fee that $U_{i}$ pays to $S_{j}$ is $C_{Sj}=UP_{sj}\times Day_{Sj}$ (unit price $UP_{sj}$ for a day of privilege service can be found on the website of $S_{j}$).

Furthermore, it is flexible for the new SP to join our system. The sub-array [$S_{j+1},...,S_{n}$] indicates the unjoined new SPs, whose values in $L_{P},L_{D}$, and $L_{C}$ are all initialized to 0. After a new SP is added to the MCC system, $U_{i}$ can use the trial privilege service of the new SP, even if the user is not registered on it. However, as $U_{i}$ wants to access higher privilege services on the new SP, the mobile user must update their access privileges.

### 4.6. Smart Contracts in Our Scheme

Our scheme uses four smart contract algorithms, namely Init_Ledger(), Check_

registration&subscription(), Read(), and Check_user_update() in Algorithms 1–4. Algorithm 1Init_Ledger() is called only once when deploying the smart contract. Algorithm 2 is called to check the validity of user registration and subscription information. Algorithm 3 is called to read the most recent value of transactions (world state) from the blockchain. Algorithm 4 is called to check the validity of the user access privilege update.
**Algorithm 2** Check_registration&subscription($PID_{i},PK_{ui},ID_{sj}^{r},PK_{sj}^{r},L,T_{1},S_{ui},S_{sj}^{r}$)1:var *U* User = $PID_{i},PK_{ui},ID_{sj}^{r},PK_{sj}^{r},L,T_{1},S_{ui},S_{sj}^{r}$;2:Take out the latest transaction $U^{*}$ about key $U.PID_{i}$ from key-value database to check the uniqueness of user identity.3:**if**$U^{*}!=Null$**then**4:    **return** Error(“user is registered");5:**end if**6:Take out the service subscription list *L* from *U*.7:**for**j=1→j=n**do** %Traverse all SPs in *L*8:    $UP_{sj}$ is one-day unit price of the access privilege $L_{P}$[*j*] on $S_{j}$;9:    $cost=UP_{sj}\times L_{D}$[*j*];10:    **if** $cost!=L_{C}$[*j*] **then**11:        **return** Error(“L is not correct or user did not pay enough");12:    **end if**13:**end for**%Finishing checking user subscription information.14:Storing the transaction *U* in the blockchain.15:Finishing user registration and subscription.16:**return** Success(“Mobile user is successfully registered !");

**Algorithm 3** Read($PID_{i}$)
1:var user User = Get_WorldState($PID_{i}$);2:%The key-value database stores the world state, which is the last transaction data about the key $PID_{i}$.3:**if**user == Null **then**4:**return**Null, fmt.Errorf(“$PID_{i}$ is not in the blockchain”);5:
**end if**
6:**return**user;


**Algorithm 4** Check_user_update($PID_{i},PK_{ui},ID_{sj}^{u},PK_{sj}^{u},L^{\prime },T_{1}^{\prime },S_{ui}^{\prime },S_{sj}^{u}$)Check user update
1:var *N* User = $PID_{i},PK_{ui},ID_{sj}^{u},PK_{sj}^{u},L^{\prime },T_{1}^{\prime },S_{ui}^{\prime },S_{sj}^{u}$;2:Take out the old transaction *O* from the blockchain.3:
**if** O==Null **then**
4:    **return** Error(“user is not registered");5:**end if**%Report an error and exit the program.6:Take out the new service subscription list $L^{\prime }$ from *N*.7:Take out the old service subscription list *L* from *O*.8:
**for**  j=1→j=n  **do**
9:    $UP_{n}$ ($UP_{o}$) is unit price of the new (old) access privilege $L_{P}^{\prime }$[*j*] ($L_{P}$[*j*]) on the SP $S_{j}$.10:    Remaining usage time $RT=L_{D}$[*j*]$-(N.T_{1}^{\prime }-O.T_{1})$;11:    **if** RT<0 **then** RT=0;12:    **end if**13:    Extension of time $t=L_{D}^{\prime }$[*j*]−RT;14:    **if** RT=0 & t==0 **then**15:        **check** $L_{P}^{\prime }$[*j*]$\overset{?}{=}0$, $L_{D}^{\prime }$[*j*]$\overset{?}{=}0$, $L_{C}^{\prime }$[*j*]$\overset{?}{=}0$;16:    **end if**17:    **if** RT=0 & t!=0 **then**18:        **check** $L_{D}^{\prime }\left[j\right]\overset{?}{=}t,L_{C}^{\prime }\left[j\right]\overset{?}{=}t\times UP_{n}$;19:    **end if**20:    **if** $RT>0\;\&\;L_{P}^{\prime }$[*j*]$<L_{P}$[*j*] **then return** Error;21:    **end if**%Cannot downgrade in unexpired service.22:    **if** $RT>0\;\&\;t==0\;\&\;L_{P}^{\prime }$[*j*]$==L_{P}$[*j*] **then**23:        **check** $L_{D}^{\prime }$[*j*]$\overset{?}{=}RT$, $L_{C}^{\prime }$[*j*]$\overset{?}{=}0$;24:    **end if**25:    **if** $RT>0\;\&\;t==0\;\&\;L_{P}^{\prime }$[*j*]$>L_{P}$[*j*] **then**26:        **check** $L_{D}^{\prime }$[*j*]$\overset{?}{=}RT$, $L_{C}^{\prime }$[*j*]$\overset{?}{=}RT\times \left(UP_{n}-UP_{o}\right)$;27:    **end if**28:    **if** $RT>0\;\&\;t!=0\;\&\;L_{P}^{\prime }$[*j*]$==L_{P}$[*j*] **then**29:        **check** $L_{D}^{\prime }$[*j*]$\overset{?}{=}RT+t$, $L_{C}^{\prime }$[*j*]$\overset{?}{=}t\times UP_{o}$;30:    **end if**31:    **if** $RT>0\;\&\;t!=0\;\&\;L_{P}^{\prime }$[*j*]$>L_{P}$[*j*] **then**32:        **check** $L_{D}^{\prime }$[*j*]$\overset{?}{=}RT+t$, $L_{C}^{\prime }$[*j*]$\overset{?}{=}RT\times \left(UP_{n}-UP_{o}\right)+t\times UP_{n}$;33:    **end if**34:**end for**%Finish checking the new service subscription list $L^{\prime }$.35:Storing the updated transaction *N* in the blockchain.36:**return** Success(“Mobile user is successfully updated !");


## 5. The Proposed Scheme

The proposed scheme consists of five phases: an initialization phase, user registration and subscription phase, authentication and authorization phase, user-friendly password update phase, and user access privilege update phase.

### 5.1. Initialization Phase

In the initialization phase, the authorized SPs will be joined into the identical blockchain network as nodes. MSP, i.e., the manager of consortium blockchain, selects an additive group of point *G* with order *q*, where *P* is a generator of *G*, and six hash secure functions $h_{0}:{\left\{0,1\right\}}^{*}\to {\left\{0,1\right\}}^{l_{0}}$, $h_{1}:{\left\{0,1\right\}}^{*}\to Z_{q}^{*}$, $h_{2}:{\left\{0,1\right\}}^{*}\to \left\{0,1,2,...,1023\right\}$, $h_{3}:{\left\{0,1\right\}}^{*}\to {\left\{0,1\right\}}^{l_{3}}$, $h_{4}:{\left\{0,1\right\}}^{*}\to {\left\{0,1\right\}}^{l_{4}}$, $h_{5}:G\to {\left\{0,1\right\}}^{l_{5}}$, where $l_{0}$, $l_{3}$, $l_{4}$, $l_{5}$ are the output bit length of the hash functions. Each SP $S_{j}$ generates a private key ${SK}_{sj}\in Z_{q}^{*}$, calculates its public key ${PK}_{sj}={SK}_{sj}\cdot P$, and stores the private key into its secret memory. $S_{j}$ registers on MSP with a public key ${PK}_{sj}$. On the website, $S_{j}$ publishes its own privilege unit price list, which records the current one-day unit price of each different privilege service. On the blockchain, all smart contracts Algorithms 1–4 are initialized and deployed. MSP publishes the parameters $\left\{G,q,P,{PK}_{sj},h_{0},h_{1},h_{2},h_{3},h_{4},h_{5}\right\}$.

### 5.2. User Registration and Subscription Phase

Each SP shares the identical ledger of the blockchain, so users can select the currently closest SP for registration and subscribing services. The Table 3 illustrates the phase of user registration and subscription.

Step 1: $U_{i}$ chooses an SP $S_{j}^{r}$ closest to the mobile device MD, selects a random number $SK_{ui}\in Z_{q}^{*}$, and generates a service subscription list *L* that records users’ desired privileges, service periods, and amount of payment. In addition, MD calculates the public key $PK_{ui}=SK_{ui}\cdot P$ and $PID_{i}=h_{5}\left(PK_{ui}\right)$, where $PID_{i}$ is the pseudonym of $U_{i}$. Then, MD generates a signature $S_{ui}=Sig(SK_{ui},PID_{i}\|T_{1}\left\|L\right)$, where $T_{1}$ is the current timestamp, and generates a NIZK proof of knowledge π:

(1) $$\begin{array}{c} \pi \leftarrow ZkPoK\{\left(SK_{ui}\right):PK_{ui}=SK_{ui}\cdot P\} \end{array}$$

Finally, $U_{i}$ transmits $\left\{PID_{i},PK_{ui},S_{ui},L,reg,T_{1},\pi \right\}$ to $S_{j}^{r}$ and the fees paid to purchase services to $S_{j}^{r}$ through a secure channel, where reg is the registration requirement.

Step 2: After receiving the message from $U_{i}$ at the time $T_{2}$, $S_{j}^{r}$ checks an inequality $|T_{2}-T_{1}|<\Delta T$, where ΔT is the maximum allowable transmission delay. If the inequality is satisfied, $S_{j}^{r}$ validates π to determine the validity of $PK_{ui}$. If it holds, $S_{j}^{r}$ confirms whether the signature $Ver(PK_{ui},S_{ui},PID_{i}\|T_{1}\|L)=1$ is correct. If it is correct, $S_{j}^{r}$ checks whether the fees paid by $U_{i}$ are equivalent to the sum of all costs in array $L_{C}$. If they are equivalent, $S_{j}^{r}$ calculates a signature $S_{sj}^{r}=Sig(SK_{sj}^{r},PID_{i}\|ID_{sj}^{r}\|PK_{ui}\|PK_{sj}^{r}\left\|L\right\|T_{1})$. Then, $S_{j}^{r}$ submits the user registration transaction $\left\{PID_{i},PK_{ui},ID_{sj}^{r},PK_{sj}^{r},L,T_{1},S_{ui},S_{sj}^{r}\right\}$ to the blockchain. Here, $T_{1}$ is the registration time of $U_{i}$.

Step 3: An overview of the transaction processing process is presented in Section 4.3. The transaction belonging to $U_{i}$ is verified by a smart contract Algorithm 2. Algorithm 2 checks whether $U_{i}$ has not registered, recalculates the entire cost, and confirms whether $C_{Sj}=UP_{sj}\times Day_{Sj}$ is correct for each SP, i.e., whether $U_{i}$ pays enough service fees for each hierarchical service. If both conditions are satisfied, one transaction that records the user identity and subscription information is stored in the blockchain. If any of the judgment conditions in Steps 2 and 3 above are not satisfied, $S_{j}^{r}$ will interrupt the session and refund service fees to users. Finally, $S_{j}^{r}$ sends {registration success} to $U_{i}$ through a secure channel.

Step 4: Upon receipt of the message, $U_{i}$ inputs $PW_{ui}$ into the mobile device MD. Then, MD chooses a random number $b_{i}$ and computes $Z_{i}=h_{0}(PID_{i}\|PW_{ui}\left\|b_{i}\right)$, $F_{i}=\left(SK_{ui}\|T_{1}\right)⊕Z_{i}$, $V=h_{3}(h_{2}(SK_{ui}\|T_{1}\|Z_{i}))$. Finally, $U_{i}$ stores $F_{i}$, *V*, $b_{i}$, $PK_{ui}$, π, and *L* into the secret memory of the mobile device.

Step 5: All SPs detecting the generation of a new transaction in blockchain ask for their deserved user subscription service fees from $S_{j}^{r}$ off chain according to the identity $ID_{sj}^{r}$ of the acceptance SP and the list *L* in the transaction.

### 5.3. Authentication and Authorization Phase

Before accessing an SP $S_{j}$, $U_{i}$ needs to implement mutual authentication with $S_{j}$. At the same time, $S_{j}$ also refers to the service subscription list *L* to determine which level of service $Lev_{Sj}$ the user can access within the validity service period $Day_{Sj}$. Table 4 illustrates the phase of authentication and authorization.

Step 1: $U_{i}$ inputs $PID_{i}$ and $PW_{ui}$ into the mobile device MD. MD computes $Z_{i}=h_{0}(PID_{i}||PW_{ui}\left|\right|b_{i})$, $SK_{ui}\|T_{1}=F_{i}⊕Z_{i}$, and $V_{0}=h_{3}(h_{2}(SK_{ui}\|T_{1}\|Z_{i}))$, and checks whether $V_{0}$ and *V* are equal. If they are not equal, MD interrupts the operation. Otherwise, MD extracts the private key $SK_{ui}$. Then, MD determines the user’s access privilege $lv\in L_{P}$ on $S_{j}$ according to the list *L*. If the privilege of $U_{i}$ has expired or is not paid for, $U_{i}$ can only access the trial privilege service, lv=0. Otherwise, $U_{i}$ can access the unexpired privilege service, $lv=Lev_{Sj}$. Next, MD generates a random number $\alpha \in Z_{q}^{*}$ and computes $X=\alpha \cdot P,H_{1}=h_{1}(X\|PID_{i}\|ID_{sj}\|T_{3}\left\|lv\right)$, $st=\alpha +H_{1}SK_{ui}$mod*q*, $M_{1}=(PID_{i}\|ID_{sj}\left\|st\|lv\right)⊕h_{4}\left(\alpha \cdot PK_{sj}\right)$, where $T_{3}$ denotes the current timestamp in $U_{i}$. Then, $U_{i}$ sends the messages $\left\{X,M_{1},T_{3}\right\}$ to $S_{j}$ through a public channel.

Step 2: Upon receipt of the message, $S_{j}$ verifies whether $|T_{4}-T_{3}|<\Delta T$, where $T_{4}$ is the timestamp when messages were received from $U_{i}$. If the inequality is satisfied, $S_{j}$ calculates $PID_{i}\|ID_{sj}\left\|st\right\|lv=M_{1}⊕h_{4}\left(SK_{sj}\cdot X\right)$. Using $PID_{i}$, the latest transaction belonging to $U_{i}$ can be queried by $S_{j}$. $S_{j}$ calls the smart contract Algorithm 3 Read (*PID_i_*) to extract the transaction $\left\{PID_{i}^{*},PK_{ui}^{*},ID_{sj}^{r*},PK_{sj}^{r*},L^{*},T_{1}^{*},S_{ui}^{*},S_{sj}^{r*}\right\}$ from blockchain. Then, $S_{j}$ obtains $PK_{ui}^{*}$ from the transaction, and verifies whether the equation $st\cdot P=X+h_{1}(X\|PID_{i}\|ID_{sj}\|T_{3}\left\|lv\right)\cdot PK_{ui}^{*}$ is satisfied. If it is satisfied, $S_{j}$ confirms that $U_{i}$ is a legal user.

Step 3: Then, $S_{j}$ checks the user’s access privilege lv and the validity period of service $Day_{Sj}$ requested by the $U_{i}$. If $\left\lfloor T_{4}-T_{1}^{*}\right\rfloor <Day_{Sj}\in L_{D}^{*}$, this indicates that the $U_{i}$ is within the validity period for using the privilege service at level $Lev_{Sj}$, $lev=Lev_{Sj}$. If $\left\lfloor T_{4}-T_{1}^{*}\right\rfloor \geq Day_{Sj}\in L_{D}^{*}$, this indicates that $U_{i}$ has expired or the privilege service ($Day_{Sj}=0$) on $S_{j}$ has not been paid for, and that only the trial privilege service, lev=0, can be used. Then, $S_{j}$ checks whether lev=lv. If the equation is satisfied, $S_{j}$ provides lev level of service for $U_{i}$. $S_{j}$ generates a random number $\beta \in Z_{q}^{*}$ and computes Y=β·P, $key=h_{3}(PID_{i}\|ID_{sj}\|X\|Y\|st\|\beta \cdot X\|lev)$, $M_{2}=MAC(key,PID_{i}\|ID_{sj}\left\|Y\|X\right\|T_{4}\left\|lev\right)$. If any of the judgment conditions in Steps 2 and 3 above are not satisfied, $S_{j}$ will interrupt the session. After that, $S_{j}$ sends $\left\{Y,M_{2},T_{4}\right\}$ to $U_{i}$ via a public channel.

Step 4: After receiving the message $\left\{Y,M_{2},T_{4}\right\}$ from $S_{j}$ at the time $T_{5}$, MD verifies whether $|T_{5}-T_{4}|<\Delta T$. If it holds, MD sets $lev^{\prime }=lv$. MD computes $key^{\prime }=h_{3}(PID_{i}\|ID_{sj}\|X\|Y\|st\|\alpha \cdot Y\|lev^{\prime })$, $M_{2}^{\prime }=MAC(key^{\prime },PID_{i}\|ID_{sj}\left\|Y\|X\right\|T_{4}\left\|lev^{\prime }\right)$. Then, MD checks whether $M_{2}^{\prime }$ matches with the received $M_{2}$. If $M_{2}^{\prime }$ holds, $U_{i}$ authenticates with $S_{j}$. Meanwhile, $U_{i}$ is also allowed to access the service with the privilege level lv on $S_{j}$ in the service period. Otherwise, $U_{i}$ fails to authenticate with the $S_{j}$.

### 5.4. User-Friendly Password Update Phase

When $U_{i}$ wants to update $PW_{i}$, the user just needs to perform the following steps in the mobile device.

Step 1: $U_{i}$ inputs $PID_{i}$ and $PW_{ui}$ into MD. Then, MD computes $Z_{i}=h_{0}(PID_{i}\|PW_{ui}\left\|b_{i}\right)$, $SK_{ui}\|T_{1}=F_{i}⊕Z_{i}$, and $V_{0}=h_{3}(h_{2}(SK_{ui}\|T_{1}\|Z_{i}))$, and checks whether $V_{0}$ and *V* are equal. If they are not equal, MD interrupts the phase of the password update. Otherwise, $U_{i}$ inputs a new password $PW^{\prime }$.

Step 2: MD computes $Z_{i}^{\prime }=h_{0}(PID_{i}\|PW_{ui}^{\prime }\left\|b_{i}\right)$, $F_{i}^{\prime }=\left(SK_{ui}\|T_{1}\right)⊕Z_{i}^{\prime }$, and $V^{\prime }=h_{3}(h_{2}(SK_{ui}\|T_{1}\|Z_{i}^{\prime }))$.

Step 3: Finally, $U_{i}$ stores $F_{i}^{\prime }$ and $V^{\prime }$ in MD to replace $F_{i}$ and *V*, respectively.

### 5.5. User Access Privilege Update Phase

The mobile user can select the closest SP to flexibly update the access privileges on n SPs in or not in the validity periods, and the mobile user can inherit the service periods that have not been used up before. Our scheme applies two arrays, *p* and *t*, to record the new privileges applied by $U_{i}$ and the extended service periods of $U_{i}$, respectively, on all SPs.

Step 1: First, $U_{i}$ chooses an SP $S_{j}^{u}$ closest to the mobile device MD, inputs its $PID_{i}$ and $PW_{ui}$, obtains $T_{1}$, $PK_{ui}$, π, and $SK_{ui}$, and sets two arrays, *p* and *t*, to determine the new levels of services desired and the extended service periods. Then, $U_{i}$ chooses the current timestamp $T_{1}^{\prime }$ and uses Algorithm 5 to obtain the new service subscription list $L^{\prime }$ with the input of *L*, $T_{1}^{\prime }$, $T_{1}$, *p*, and *t*. After that, $U_{i}$ calculates $S_{ui}^{\prime }=Sig(SK_{ui},PID_{i}\|T_{1}^{\prime }\left\|L^{\prime }\right)$. Finally, $U_{i}$ submits $\left\{PID_{i},PK_{ui},S_{ui}^{\prime },L^{\prime },renew,T_{1}^{\prime },\pi \right\}$ and service fees to $S_{j}^{u}$ through the secure channel, where renew is the updated requirement.

Step 2: After receiving the message, $S_{j}^{u}$ checks the inequality $|T_{6}-T_{1}^{\prime }|<\Delta T$, where $T_{6}$ denotes the timestamp of when $S_{j}^{u}$ received the updated message. If the inequality is satisfied, $S_{j}^{u}$ validates π to determine the validity of $PK_{ui}$. If it holds, $S_{j}^{u}$ confirms whether the signature $Ver(PK_{ui},S_{ui}^{\prime },PID_{i}\|T_{1}^{\prime }\|L^{\prime })=1$ is correct. If it is correct, $S_{j}^{u}$ checks whether fees paid by $U_{i}$ are equivalent to the sum of all costs in array $L_{C}^{\prime }$. If they are equivalent, $S_{j}^{u}$ calculates $S_{sj}^{u}=Sig(SK_{sj}^{u},PID_{i}\|ID_{sj}^{u}\|PK_{ui}\|PK_{sj}^{u}\|L^{\prime }\left\|T_{1}^{\prime }\right)$. Then, $S_{j}^{u}$ submits update transaction $\left\{PID_{i},PK_{ui},ID_{sj}^{u},PK_{sj}^{u},L^{\prime },T_{1}^{\prime },S_{ui}^{\prime },S_{sj}^{u}\right\}$ to the blockchain.

Step 3: The overview of the transaction processing process is presented in Section 4.3. The update transaction belonging to the $U_{i}$ is verified by a smart contract Algorithm 4. Algorithm 4 checks whether the $U_{i}$ has been registered and whether the cost $L_{C}^{\prime }$ of the $L^{\prime }$ is set correctly. If both conditions are satisfied, one transaction that records the user update information is stored in the blockchain. If any of the above conditions in Steps 2 and 3 are not satisfied, $S_{j}^{u}$ interrupts the session and refunds fees to users. Finally, $S_{j}^{u}$ sends {updatesuccess} to $U_{i}$ through the secure channel.

Step 4: After receiving the message, $U_{i}$ computes $Z_{i}=h_{0}(PID_{i}\|PW_{ui}\left\|b_{i}\right)$, $F_{i}^{*}=\left(SK_{ui}\|T_{1}^{\prime }\right)⊕Z_{i}$, and $V^{*}=h_{3}(h_{2}(SK_{ui}\|T_{1}^{\prime }\|Z_{i}))$. Finally, $U_{i}$ stores $F_{i}^{*}$, $V^{*}$, and $L^{\prime }$ in the secret memory of MD to replace $F_{i}$, *V*, and *L*, respectively.

Step 5: All SPs, detecting the generation of a new transaction in blockchain ask for their deserved user update service fees from $S_{j}^{u}$ off chain according to the identity $ID_{sj}^{u}$ of the acceptance SP and the list $L^{\prime }$ in the transaction.
**Algorithm 5** User updating the service subscription list *L***Input:** Old service subscription list *L*, Current timestamp $T_{1}^{\prime }$, Previous registration time $T_{1}$, New access privileges *p*[] set by the user, Extension of periods *t*[] set by the user.**Output:** New service subscription list $L^{\prime }$.1:Update user’s access privileges and service periods on all n SPs in turn.2:**for**j=1→j=n**do**3:    remaining service periods $RT=L_{D}$[*j*]$-(T_{1}^{\prime }-T_{1})$;4:    **if** RT<0 **then** RT=0;5:    **end if**6:    $UP_{n}$ ($UP_{o}$) is unit price of the new (old) access privilege *p*[*j*] ($L_{P}$[*j*]) on the SP $S_{j}$.7:    **if** RT=0 **then** %$U_{i}$ has expired or the service on $S_{j}$ has not been bought.8:        **if** t[j]==0 **then** %$U_{i}$ do not buy the service period on $S_{j}$ this time.9:           $L_{P}^{\prime }\left[j\right]=0,L_{D}^{\prime }\left[j\right]=0$, $L_{C}^{\prime }\left[j\right]=0$;10:        **end if**11:        **if** t[j]!=0**then** %$U_{i}$ buy the service period on $S_{j}$.12:           $L_{P}^{\prime }\left[j\right]=p\left[j\right],L_{D}^{\prime }\left[j\right]=t\left[j\right],L_{C}^{\prime }\left[j\right]=t\left[j\right]\times UP_{n}$;13:        **end if**%$U_{i}$ buy new privilege services.14:    **end if**15:    **if** RT>0 **then** %$U_{i}$ has not expired on $S_{j}$.16:        **if** $p\left[j\right]<L_{P}\left[j\right]$ **then return** Error;17:        **end if**%Privileges cannot be reduced during service periods.18:        **if** t[j]==0 **then** %Do not extend the use period on $S_{j}$.19:           **if** $p\left[j\right]==L_{P}\left[j\right]$ **then** $L_{P}^{\prime }\left[j\right]=L_{P}\left[j\right],L_{D}^{\prime }\left[j\right]=RT,L_{C}^{\prime }\left[j\right]=0$;20:           **end if**%Update the use period on $S_{j}$.21:           **if** $p\left[j\right]>L_{P}\left[j\right]$ **then** $L_{P}^{\prime }\left[j\right]=p\left[j\right],L_{D}^{\prime }\left[j\right]=RT,L_{C}^{\prime }\left[j\right]=RT\times \left(UP_{n}-UP_{o}\right)$;22:           **end if**%Increase privileges needed to make up the difference.23:        **end if**24:        **if** t[j]!=0 **then** %Extend the use period on $S_{j}$.25:           **if** $p\left[j\right]==L_{P}\left[j\right]$ **then** $L_{P}^{\prime }\left[j\right]=L_{P}\left[j\right],L_{D}^{\prime }\left[j\right]=RT+t\left[j\right],L_{C}^{\prime }\left[j\right]=t\left[j\right]\times UP_{o}$;26:           **end if**%Continue to buy the previous privilege service.27:           **if** $p\left[j\right]>L_{P}[j$] **then** $L_{P}^{\prime }\left[j\right]=p\left[j\right],L_{D}^{\prime }\left[j\right]=RT+t\left[j\right],L_{C}^{\prime }\left[j\right]=RT\times \left(UP_{n}-UP_{o}\right)+t\left[j\right]\times UP_{n}$;28:           **end if**%Increase privileges needed to make up the difference.29:        **end if**30:    **end if**31:**end for**32:**return**$\left(L^{\prime }\right)$;

## 6. Example

To illustrate our algorithms, we give an example that implements smart contracts Algorithms 1–4 on the blockchain platform. Since Algorithm 1 is only called once in the initialization phase, we mainly present the realization of Algorithms 2–4, which are used in the user registration and subscription phase, authentication and authorization phase, and user access privilege update phase.

### 6.1. Blockchain Platform

**Figure 4:**
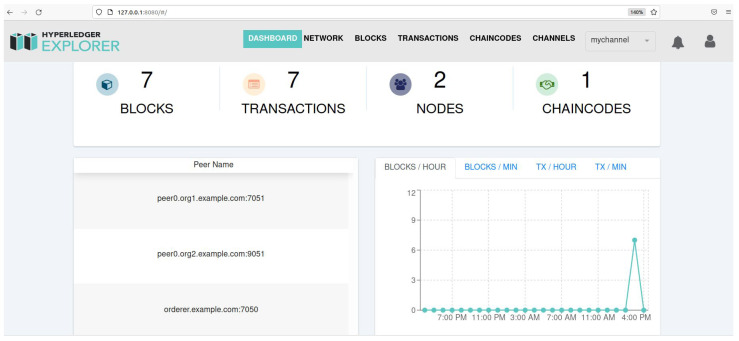
Overview of our blockchain: two peers, an order, and a chaincode including four smart contracts.

**Figure 5:**
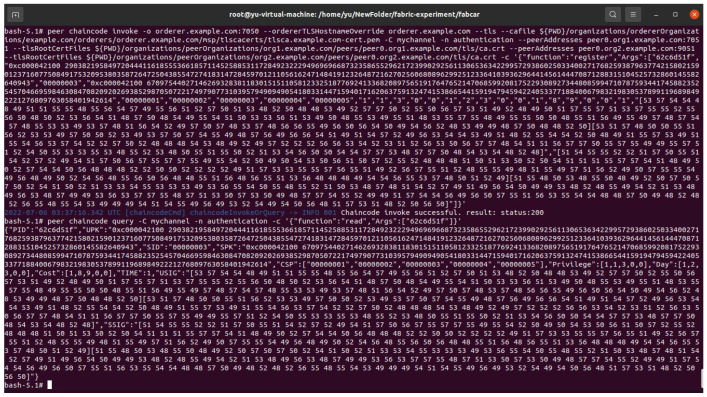
The smart contract of Check_registration&subscription() invoked in the virtual machine.

**Figure 6:**
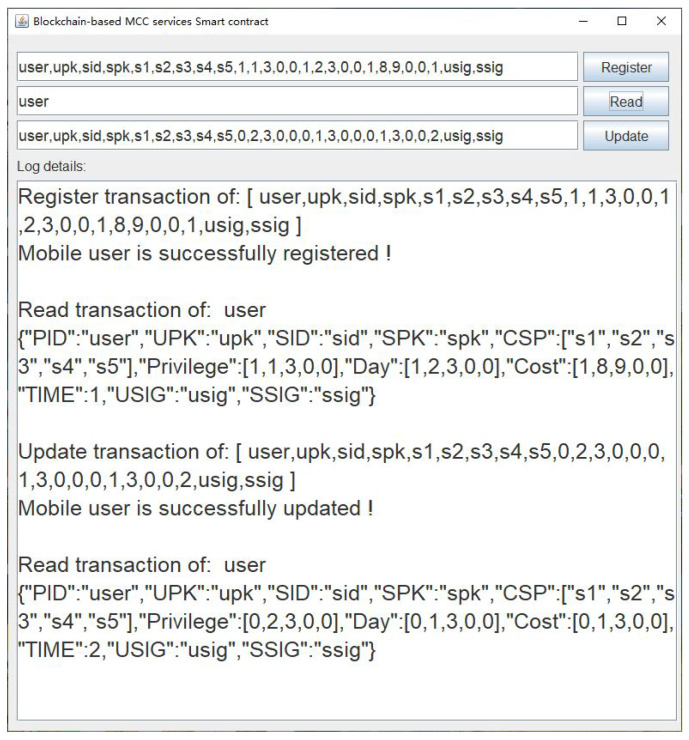
The smart contracts invoked in the java application: the “Register” button is clicked, the smart contract Algorithm 2 Check_registration&subscription() is invoked through Fabric-sdk-java, and registration information is entered in the text box as input algorithm parameters. Then, the smart contract returns the running result. The same is true for the button “Read" of Algorithm 3 Read() and the button “Update” of Algorithm 4 Check_user_update().

In the proposed scheme, the blockchain platform is implemented on Hyperledger Fabric 2.3, with the functions of ledger initialization, user registration, read transaction, and user update. The four functions are achieved by the smart contract. We set up two peer nodes that store transaction data independently and an order node that can publish transactions in the blockchain. A Hyperledger Fabric-sdk-java was used to operate the transaction and invoke the smart contract. The platform device information is shown in Table 5, and the browser page of Hyperledger Fabric shows an overview of our blockchain in Figure 4. In addition, Figure 5 indicates the invoked result of the registration Algorithm 2 on the virtual machine. However, for explaining our experiment more clearly, we used a java application, as shown in Figure 6, to invoke smart contract algorithms from the blockchain. In the java application, we used the string to replace the static encoding result of identities, public keys, and signatures, to focus on the dynamic check of the service subscription list.

### 6.2. Implementation of User Registration and Subscription

In the registration phase, as shown in Table 6, $U_{i}$ chooses $L_{S}$ = [$S_{1},S_{2},S_{3},S_{4},S_{5}$], where [$S_{4},S_{5}$] are unjoined SPs. In the list $L_{1}$, $U_{i}$ sets the privileges $L_{P}=\left[1,1,3,0,0\right]$ to be accessed and the days $L_{D}=\left[1,2,3,0,0\right]$ to use the privilege service, and $S_{1}$ to $S_{5}$ set the unit price UP of privilege services to be accessed as [1,4,3,0,0]. According to the calculation formula $C_{Sj}=UP\times Day_{Sj}$, the cost array $L_{C}$ is set to [1,8,9,0,0]. The arrays $L_{P},L_{D},L_{C}$ of the unjoined new SPs $S_{4},S_{5}$ should be set to 0 in the $L_{1}$. In this experiment, the field TIME in the transaction of the registration phase was set to the integer 1 (TIME needs to be a timestamp in the practical application).

Then, $U_{i}$ sends service fees 18 and registration information that contains $L_{1}$ to $S_{3}$, which is currently close to $U_{i}$. $S_{3}$ checks π, $S_{ui}$, and whether the sum of $L_{C}$ is equal to 18. If it holds, $S_{3}$ signs the registration information and submits it to the blockchain by invoking the smart contract Algorithm 2, as shown in Figure 6. Algorithm 2 checks whether the user has not registered and whether $L_{1}$ is correct. The returned result of invoking Algorithm 2 was “Mobile user is successfully registered !”, which indicates that the service fees paid for each SP passed the check, and that the registration transaction has been written into the blockchain. In addition, we successfully queried the registration transaction with the key “user” in the CouchDB. Finally, $S_{1}$ and $S_{2}$ can ask for their deserved service fees 1 and 8 from $S_{3}$ off chain according to list $L_{1}$ in the transaction.

### 6.3. Implementation of User Access Privilege Update

In the user access privilege update phase, $U_{i}$ updated the service subscription list $L_{1}$ submitted in the registration phase. As shown in Table 7, the settings of SPs in the new service subscription list $L_{2}$ remained unchanged. The field TIME in the transaction of the update phase was set to the integer 2. The change in the field TIME in two phases indicated that one day has passed, and $U_{i}$ extended the usage time of the privilege service on $S_{3}$ by 1 day. Thus, the days $L_{D}$ to use the privilege service should be set to [0,1,3,0,0]. Since the time of using the service of $S_{1}$ was 0, the user’s privilege to access $S_{1}$ should be set to 0. $U_{i}$ updated the user’s privilege on $S_{2}$ to 2, and the privilege on $S_{3}$ remained as 3. In brief, the privileges $L_{P}$ = [0,2,3,0,0] were updated in the list $L_{2}$. The unit price UP of privilege services access in the update phase was set to [0,5,3,0,0]. According to Algorithm 5, $U_{i}$ calculated the costs on $S_{1}$, $S_{2}$, and $S_{3}$, which were 0×0, (5−4)×1, and 3×1. The service on $S_{2}$ is only upgraded, and the price difference needs to be made up. $U_{i}$ pays for one day to extend the service period on $S_{3}$. Therefore, the cost array $L_{C}$ should be set to [0,1,3,0,0]. Similar to the registration stage, the arrays $L_{P},L_{D},L_{C}$ of $S_{4},S_{5}$ should be set to 0.

Then, $U_{i}$ sends service fees 4 and update information that contains $L_{2}$ to $S_{1}$, which is currently close to $U_{i}$. $S_{1}$ checks π, $S_{ui}^{\prime }$, and whether the sum of $L_{C}$ is equal to 4. If it holds, $S_{1}$ signs the update information and submits it to the blockchain by invoking the smart contract Algorithm 4, as shown in Figure 6. Algorithm 4 checks whether the user has registered and whether $L_{2}$ is correct. The returned result of Algorithm 4 was “Mobile user is successfully updated !”, which indicates that the amount paid for each SP passed the check, and that the update transaction has been written into the blockchain. In addition, we successfully queried the update transaction with the key “user” in the CouchDB. Finally, $S_{2}$ and $S_{3}$ can ask for their deserved service fees 1 and 3 from $S_{1}$ off chain according to list $L_{2}$ in the transaction.

## 7. Security Analysis of the Proposed Scheme

This section proves that the proposed scheme satisfies the security requirements defined in Section 3 using the random oracle model. Since the authentication phase is executed in an insecure public channel, and other phases are implemented in the secure channel, this section shows the resistance to security threats in the authentication phase.

**Security Model.** Following the literature [11,39,46] a security model was designed for the proposed scheme, which was demonstrated by a game played by a probabilistic polynomial time (PPT) Turing machine *ℑ* and a PPT adversary A. We used the instances $\prod_{U}^{S}$ and $\prod_{S}^{S}$ to represent the mobile user oracle and the SP oracle, respectively, in session S. The adversary A was allowed to perform the following attack capabilities:

1. $RegisterU_{i}-Oracle:$A issues this inquiry to simulate registering as a legal mobile user $U_{i}$ with the identity $PID_{i}$. *ℑ* generates the access privilege lv and the private key and public key of $U_{i}$, records them in the list $L_{U}$, and returns $PID_{i}$ and lv to A.
2. $RegisterS_{j}-Oracle:$A issues this inquiry to simulate registering as a legal SP $S_{j}$ with the identity $ID_{sj}$. *ℑ* generates the access privilege lev and the private key and public key of $S_{j}$, records them in the list $L_{S}$, and returns $ID_{sj}$ and lev to A.
3. $Send-Oracle(\vartheta ,S,\vartheta^{\prime },M):$A issues this inquiry to simulate that the participant $\vartheta^{\prime }$ transmits the message M to the oracle $\prod_{\vartheta }^{S}$, and *ℑ* returns an answer specified by the protocol to A.
4. Execute-Oracle:A issues this inquiry to simulate using all passive attacks, and *ℑ* returns all messages transmitted between $U_{i}$ and $S_{j}$.
5. There are three corruption queries:
  (a) $Corrupt(PID_{i},PW_{i}):$A issues this inquiry to simulate the password leakage attack, and *ℑ* returns the mobile user password $PW_{i}$ to A.
  (b) $Corrupt(PID_{i},MD_{i}):$A issues this inquiry to simulate the mobile device $MD_{i}$ loss attack, and *ℑ* returns secret parameters stored in $MD_{i}$ to A.
  (c) $Corrupt(S_{j}):$A issues this inquiry to simulate the SP compromise attack.

**Definition** **1**. *Matching sessions: If the session in $\prod_{U}^{S}$ and the session in $\prod_{S}^{S^{\prime }}$ are the same session $S=S^{\prime }$, the peer identity of $\prod_{S}^{S^{\prime }}$ is $Pid_{S}=U$, the peer identity of $\prod_{U}^{S^{\prime }}$ is $Pid_{U}=S$, and two instances have both been accepted, the two session S and $S^{\prime }$ are said to be matching.*

**Definition** **2**. *Security authentication protocol: The secure authentication scheme needs to satisfy the following properties:*

1. *$\prod_{U}^{S}$ and $\prod_{S}^{S^{\prime }}$ are matching sessions, and two instances accept each other.*
2. *The probability that A is accepted as $\prod_{S}^{S}$ by $\prod_{U}^{S}$ is negligible.*
3. *The probability that A is accepted as $\prod_{U}^{S}$ by $\prod_{S}^{S}$ is negligible.*

**Definition** **3**. Discrete logarithm (DL) problem hypothesis: Given that X=α·P, $\alpha \in Z_{q}^{*}$, X∈G, it is infeasible to calculate α.

MAC-Game: there exist two participants, the challenger and the MAC oracle $\prod_{M}$, where $\prod_{M}$ possesses the key. The challenger is able to input any messages to the MAC oracle $\prod_{M}$ to obtain an MAC value as many times as needed. The probability for the adversary to win the MAC-Game is assumed to be $Pr_{adv}\left[MAC\right]$. The MAC-Game has the following steps:

1. The adversary transmits two messages $m_{0}$ and $m_{1}$ to $\prod_{M}$.
2. $\prod_{M}$ selects a random number b∈{0,1}. If b=0 is selected, $\prod_{M}$ returns the MAC value $MAC(key,m_{0})$ to A. If b=1 is selected, $\prod_{M}$ returns the MAC value $MAC(key,m_{1})$ to A.
3. The adversary speculates on the value of *b* and give a $b^{\prime }$. If $b=b^{\prime }$, A can win the MAC-Game.

### 7.1. Formal Security Analysis

In this section, the adversary A and Turing machine *ℑ* play a game to show that if A can successfully impersonate a mobile user or SP to pass authentication with a non-negligible probability, then a PPT *ℑ* can be constructed to solve the potential hard problems with a non-negligible probability using the abilities of A.

Our proposed protocol is reviewed as below:

1. $U_{i}\to S_{j}:$$M_{1}=\left\{X,M_{1},T_{3}\right\}.$
2. $S_{j}\to U_{i}:$$M_{2}=\left\{Y,M_{2},T_{4}\right\}.$

**Lemma** **1** (Secure Mobile User Authentication). *In our proposed scheme, if $\prod_{S}^{S}$ is accepted, solving the DL problem is infeasible, all hash functions are ideal random functions, and the probability that a PPT adversary A forges a legitimate mobile user authentication message is negligible.*

**Proof.**  We assume that a PPT adversary A is able to forge a legitimate mobile user authentication message to pass authentication with a non-negligible probability. Afterwards, a PPT Turing machine *ℑ* is able to win the DL problem with a non-negligible probability by using the abilities of A. The probability that the advantage for A wins the DL problem is assumed to be $Pr_{adv}\left[DL\right]$. □

Giving an example of a DL problem, the Turing machine *ℑ* needs to calculate the $\alpha \in Z_{q}^{*}$ using the known values X=α·P and *P*. In addition, all oracle queries of A must be answered by *ℑ* to simulate an environment where A cannot distinguish from the real proposed scheme. Therefore, all initialization parameters $\left\{G,q,P,{PK}_{sj},h_{0},h_{1},h_{2},h_{3},h_{4},h_{5}\right\}$ should be generated and published by *ℑ*. The private key of the challenger $PID_{c}$ is assumed to be $SK_{c}$, and *ℑ* answers all oracle queries of A as follows:

1. $H_{i}\left(m_{i}\right)$: In this query, *ℑ* maintains a list $L_{hi}$ initialized to empty, and i = 0, 1, 2, 3, 4, 5. After receiving the message $m_{i}$, *ℑ* inspects whether [$m_{i},h$] is kept in $L_{hi}$. If it is kept, *ℑ* returns *h* to A. Otherwise, *ℑ* randomly generates a number *h*, maintains the tuple $\left[m_{i},h\right]$ in the list $L_{hi}$, and returns *h* to A.
2. $RegisterU_{i}-Oracle$: In this query, *ℑ* maintains a list $L_{U}$ initialized to empty. *ℑ* inspects if [$PID_{i},PK_{ui},SK_{ui},lv$] is kept in $L_{U}$. If it is kept, *ℑ* returns $PID_{i}$ and lv to A. Otherwise, *ℑ* operates as follows:
  (a) If $PID_{i}$ = $PID_{c}$, *ℑ* sets $SK_{ui}=⊥$ and obtains the public key $PK_{ui}$ and access privilege lv from the mobile user oracle $\prod_{U}^{S}$. Then, *ℑ* maintains the tuple [$PID_{i},PK_{ui},SK_{ui},lv$] in the list $L_{U}$, and returns $PID_{i}$ and lv to A.
  (b) If $PID_{i}\neq PID_{c}$, *ℑ* generates an access privilege lv, randomly selects a number $SK_{ui}\in Z_{q}^{*}$, and computes the public key $PK_{ui}=SK_{ui}\cdot P$. Then, the tuple [$PID_{i},PK_{ui},\;SK_{ui},lv$] is maintained in the list $L_{U}$, and *ℑ* returns $PID_{i}$ and lv to A.
3. $Send-Oracle(U_{i},S,S_{j},M):$ In this query, A transmits the first message $M_{1}$ to *ℑ*. $M_{1}$ is decrypted by *ℑ* to obtain $PID_{i}$, $PK_{ui}$ and lv. Then, *ℑ* is executed following the specification of the proposed protocol and returns $M_{2}$.
4. $Send-Oracle(S_{j},S,U_{i},M):$ In this query, A first checks whether $PID_{i}$=$PID_{c}$ is satisfied. If not, *ℑ* is executed following the specification of the proposed protocol and returns $M_{1}$ to A. Otherwise, *ℑ* asks $\prod_{U}^{S}$ for $M_{1}$, and then returns $M_{1}$ to A.
5. Execute-Oracle: In this query, *ℑ* returns all messages transmitted between $U_{i}$ and $S_{j}$ to A.
6. $Corrupt(ID_{i},PW_{i})$: In this query, *ℑ* asks $\prod_{U}^{S}$ for the password $PW_{i}$ and returns it to A.
7. $Corrupt(ID_{i},MD_{i})$: In this query, *ℑ* asks $\prod_{U}^{S}$ for the secret parameters in mobile devices $MD_{i}$ and returns it to A.
8. $Corrupt(S_{j}):$ In this query, *ℑ* returns the private key $SK_{sj}$ of the SP $S_{j}$ to A.

If A can successfully falsify an authentication message $M_{1}=\left\{X,M_{1},T_{3}\right\}$ sent to *ℑ*, the adversary will pass user authentication, where $M_{1}=(PID_{i}\|ID_{sj}\left\|st\|lv\right)⊕h_{4}\left(\alpha \cdot PK_{sj}\right)$, and $st=\alpha +H_{1}SK_{ui}$mod *q*. Due to the forking lemma [47], a counterfeit authentication message $M_{1}=\left\{X,M_{1}^{\prime },T_{3}\right\}$ is forged by A using the repeat of the simulation with the value of $h_{4}\left(\alpha \cdot PK_{sj}\right)$. Therefore, two Equations (2) and (3) are shown as follows:

(2) $$st=\alpha +H_{1}SK_{ui}\;mod\;q$$

(3) $$st^{\prime }=\alpha +H_{1}^{\prime }SK_{ui}\;mod\;q$$

Calculating the two equations, the following Equation (4) is obtained:

(4) $$st-st^{\prime }=\left(H_{1}-H_{1}^{\prime }\right)SK_{ui}$$

According to the above analysis, $\left(st-st^{\prime }\right){\left(H_{1}-H_{1}^{\prime }\right)}^{-1}$ is the solution of the DL problem. A further analysis of the probability is shown below. If A successfully forges a legitimate authentication message with the non-negligible probability η, *ℑ* can solve the DL problem. If A cannot achieve forgery, γ represents the probability that A wins the DL problem. Hence, the probability that the advantage for A wins the DL problem is computed as the following Equation (5), similar to that of the paper [46]:

(5) $$\begin{array}{cc} Pr_{adv}\left[DL\right] & =\frac{1}{n_{s}}\cdot \left(\eta \cdot 1+\left(1-\eta \right)\cdot \gamma \right)+\frac{n_{s}-1}{n_{s}}\cdot \gamma \\ \; & =\frac{\eta +\gamma \cdot (n_{s}-\eta )}{n_{s}} \end{array}$$

where $n_{s}$ represents the number of queries sent by A. Since the probability η is non-negligible and the probability γ is negligible, $Pr_{adv}\left[DL\right]$ is also non-negligible. Thus, *ℑ* has a non-negligible probability of winning the DL problem using the abilities of A. However, this is a contradiction to our assumption. In our scheme, there is no PPT A that can successfully forge a legal authentication message of mobile users with a non-negligible probability.

**Lemma** **2** (Secure SP Authentication). *In our proposed scheme, if $\prod_{U}^{S}$ is accepted, solving the DL problem is infeasible, all hash functions are ideal random functions, and the probability that a PPT adversary A forges a legal SP authentication message is negligible.*

**Proof.**  We assume that a PPT adversary A is able to forge a legitimate SP authentication message to pass authentication with a non-negligible probability. Afterwards, a PPT Turing machine *ℑ* is able to win the latent game of MAC (MAC-Game) with a non-negligible probability by using the abilities of A without knowing the key. □

All oracle queries of A must be answered by *ℑ* to simulate an environment where A cannot distinguish from the real proposed scheme. Therefore, all initialization parameters need to be generated and published by *ℑ*, except the private key $SK_{cs}$ of the challenger $ID_{cs}$. The oracle queries $H_{i}\left(m_{i}\right)$, $RegisterU_{i}-Oracle$, Reveal-Oracle, and Execute-Oracle are answered by *ℑ* operating in the proof of Lemma 1, and *ℑ* answers other oracle queries of A as follows:

1. $RegisterS_{j}-Oracle$: In this query, *ℑ* maintains a list $L_{S}$ initialized to empty. *ℑ* inspects if [$ID_{sj},PK_{sj},SK_{sj}$, lev] is kept in $L_{S}$. If it is kept, *ℑ* returns $ID_{sj}$ and lev to A. Otherwise, *ℑ* operates as follows:
  (a) If $ID_{sj}$ = $ID_{cs}$, *ℑ* sets $SK_{sj}=⊥$ and obtains the public key $PK_{sj}$ and the access privilege lev from the SP oracle $\prod_{S}^{S}$. Then, *ℑ* maintains the tuple [$ID_{sj},PK_{sj},SK_{sj}$, lev] in the list $L_{S}$ and returns $ID_{sj}$ and lev to A.
  (b) If $ID_{sj}\neq ID_{cs}$, *ℑ* randomly selects a number $SK_{sj}\in Z_{q}^{*}$, sets lev, and computes the public key $PK_{sj}=SK_{sj}\cdot P$. Then, *ℑ* maintains the tuple [$ID_{sj},PK_{sj},SK_{sj}$, lev] in the list $L_{S}$ and returns $ID_{sj}$ and lev to A.
2. $Send-Oracle(U_{i},S,S_{j},M)$: In this query, A transmits the first message $M_{1}$ to *ℑ*. Then, *ℑ* is executed following the specification of the proposed protocol and returns $M_{2}$. After receiving $M_{2}$ from A, *ℑ* asks $\prod_{M}$ to test and check the received MAC value in $M_{2}$, and returns the result of verification.
3. $Send-Oracle(S_{j},S,U_{i},M)$: In this query, A first checks whether $ID_{sj}$ = $ID_{cs}$ is satisfied. If not, *ℑ* is executed following the specification of the proposed protocol and returns $M_{1}$ to A. Otherwise, *ℑ* terminates the MAC-Game.
4. $Corrupt(S_{j})$: In this query, *ℑ* checks whether $ID_{sj}$ = $ID_{cs}$ is satisfied. If it is not satisfied, *ℑ* returns the private key $SK_{sj}$ of the SP $S_{j}$ to A. Otherwise, *ℑ* terminates the MAC-Game.

If A can successfully falsify an SP authentication message $M_{2}=\left\{Y,M_{2},T_{4}\right\}$ sent to *ℑ*, the adversary will pass SP authentication, where $M_{2}=MAC(key,PID_{i}\|ID_{sj}\left\|Y\|X\right\|T_{4}\left\|lev\right)$. After receiving $M_{2}=\left\{Y,M_{2},T_{4}\right\}$, *ℑ* transmits the message $m_{0}=\{PID_{i}\|ID_{sj}\left\|Y\|X\right\|T_{4}\left\|lev\right\}$ and a random $m_{1}$ whose bit length is equal to $m_{0}$ to $\prod_{M}$. $\prod_{M}$ returns an MAC value $MAC(key,m_{b})$ to *ℑ*. According to the value of $M_{2}$, *ℑ* is able to inspect whether the value of $MAC(key,m_{b})$ is $MAC(key,m_{0})$ or $MAC(key,m_{1})$ to obtain b=0 or b=1. If A successfully forges a legitimate SP authentication message with a non-negligible probability η, *ℑ* can win the MAC-Game. If A cannot achieve forgery, *ℑ* has a probability of 1/2 to win the MAC-Game. Hence, the probability that the advantage for A wins the MAC-Game is computed as the following formula 6, similar to that of the paper [46]:

(6) $$\begin{array}{cc} Pr_{adv}\left[MAC\right] & =\frac{1}{n_{s}}\cdot \left(\eta \cdot 1+\left(1-\eta \right)\cdot \frac{1}{2}\right)+\frac{n_{s}-1}{n_{s}}\cdot \frac{1}{2}-\frac{1}{2}\\ \; & =\frac{\eta }{2n_{s}} \end{array}$$

Since the probability η is non-negligible, $Pr_{adv}\left[MAC\right]$ is also non-negligible. Thus, *ℑ* has a non-negligible probability of winning the MAC-Game using the abilities of A. However, this is a contradiction to our assumption. In our scheme, there is no PPT A that can successfully forge a legal SP authentication message with a non-negligible probability.

**Theorem** **1**. *If: (A) $\prod_{U}^{S}$ and $\prod_{S}^{S}$ has been accepted, (B) solving the DL problem is infeasible, and (C) all hash functions $h_{i}$ i = 0,…,5 are ideal random functions, the proposed scheme is secure.*

**Proof.**  Lemma 1 and Lemma 2 can confirm that there is no PPT A that is able to successfully forge a legitimate mobile user or SP authentication message with a non-negligible probability if solving the DL problem is infeasible and MAC is the ideal random function. Thus, our proposed scheme is secure based on Definition 2. □

### 7.2. Informal Security Analysis

#### 7.2.1. Single Registration

A mobile user, interested in multiple services, just needs to selects the arbitrary (nearest) SP $S_{j}^{r}$ to submit the identity, subscription information, and service fees. (1) $S_{j}^{r}$ validates π to determine that the user is the owner of $SK_{ui}$, ensuring the validity of the user identity. In addition, the smart contract Algorithm 2 checks the uniqueness of the user identity by reading the information about $PID_{i}$ on the blockchain. (2) Algorithm 2 inspects the correctness of the user’s subscription information in the list *L* and decides whether to store the transaction in the blockchain to accomplish the single registration for the mobile user. (3) Other SPs can ask for their deserved user registration service fees from $S_{j}^{r}$ off chain according to the identity $ID_{sj}$ of the acceptance SP and the list *L* in the transaction. The three phases are secure under the ZkPoK protocol, the automatic and transparent smart contract, and the tamper-proof distributed ledger.

#### 7.2.2. Mutual Authentication

Based on Theorem 1, there is no PPT adversary A that is able to successfully forge a legitimate mobile user or SP authentication message with a non-negligible probability if solving the DL problem is infeasible and MAC is the ideal random function. Hence, mutual authentication between mobile users and SPs can be guaranteed.

#### 7.2.3. User Anonymity and Un-Traceability

To provide user anonymity on the blockchain, the pseudonym $PID_{i}=h_{5}\left(PK_{ui}\right)$ is used in the transaction. $PID_{i}$ is only bound to the user’s subscription information, and not the real identity. In the public channel, $PID_{i}$ is protected by $h_{4}\left(\alpha \cdot PK_{sj}\right)$. The random number α will change for each authentication session. $PK_{sj}$ is calculated by $SK_{sj}$, but $SK_{sj}$ is confidentially stored in the memory of SP. After intercepting the message $\left\{X,M_{1},T_{3}\right\}$, A either guesses the value of α or solves the DL problem. Both operations are practically infeasible. Thus, A cannot obtain the mobile user’s true identity and track the communication process of a mobile user.

#### 7.2.4. Two-Factor Security

There exist two security factors in our scheme. One is PW, and the other is the mobile device. When only having the password without a mobile device, the adversary cannot accurately generate each cryptographic parameter to forge the mobile user authentication message. On the other hand, when the mobile device is lost or stolen by an adversary, then A is able to extract the secret parameters from the mobile device. During offline dictionary attacks, the password space $|D_{PW}|$ is divided into 1024 candidate passwords $|D_{PW}/1024|$ [48,49]. Consequently, A is still unable to obtain the correct password.

#### 7.2.5. Resistance to Reply Attack

The timestamp and challenge0-response mechanism are used in our proposed scheme to prevent the replay attack. Mobile user authentication message $\left\{X,M_{1},T_{3}\right\}$ and SP authentication message $\left\{Y,M_{2},T_{4}\right\}$ are protected by timestamps $T_{3}$ and $T_{4}$. After an SP or the mobile user receives the timestamp, the validity of $T_{3}$ or $T_{4}$ will be checked. Hence, the user and SP will only accept each other in the current session.

#### 7.2.6. Resistance to Wrong Password Login/Update Attack

If A enters the wrong password $PW^{\prime }$, the mobile device will calculate a wrong $V_{0}^{\prime }=h_{3}(h_{2}(SK_{ui}^{\prime }\|T_{1}^{\prime }\|Z_{i}^{\prime }))$, and check whether $V_{0}^{\prime }$ and *V* are equal. This equation is untenable, and the wrong password login/update will be refused.

#### 7.2.7. Hierarchical Access Control

The service provided by each SP is divided into several levels, e.g., ‘Bronze’, ‘Silver’, and ‘Gold’. A mobile user can subscribe to different levels of hierarchical services on multiple SPs. The service subscription list *L* records service levels (access privileges) and service periods that users want to subscribe to. Users only need to determine service levels that they are interested in and the service usage time in *L*, and submit *L* to the blockchain for the inspection of Algorithm 2. Users are not required to interact with SPs in advance. In the authentication and authorization phase, the SP looks up the mobile user’s service level (access privilege) and service period in *L* to determine which level of service the user can access.

#### 7.2.8. Access within Limits of Permission

In the authentication and authorization phase, $U_{i}$ sends the access privilege lv to SP. Then, SP reads the service subscription list *L* from the blockchain and checks the user’s access privilege lv and the validity period of service. If $U_{i}$ has not expired on the $S_{j}$ with the privilege $Lev_{Sj}$, $\left\lfloor T_{4}-T_{1}^{*}\right\rfloor <Day_{Sj}\in L_{D}^{*}$, SP sets $lev=Lev_{Sj}$ to allow users to access privilege services. If $U_{i}$ has expired on the $S_{j}$, $\left\lfloor T_{4}-T_{1}^{*}\right\rfloor \geq Day_{Sj}\in L_{D}^{*}$, SP sets lev=0 to provide the free trial service. More importantly, SP needs to check whether lv=lev. If they are not equal, this indicates that $U_{i}$ is trying to access beyond its authority or has expired, and the SP will reject the access request and interrupt the session. In addition, if the SP sets the wrong privilege lev, the session will also be interrupted.

#### 7.2.9. Efficient and Flexible Update User Access Privilege

If $U_{i}$ has expired or the service has not been purchased on an SP, the mobile user can subscribe to all levels of services. Even if $U_{i}$ is still within the validity period of the service on an SP, the mobile user can also subscribe to the service period of new privilege services, and the user only needs to make up the price difference without having to fully pay again. Moreover, Algorithm 5 will set up the user’s new access privileges and service periods on all SPs simultaneously in order to generate a correct new service subscription list $L^{\prime }$. $L^{\prime }$ is sent to $S_{j}^{u}$ and stored in a new transaction after inspection. Hence, our scheme only needs $U_{i}$ to send an update request, and the user’s service levels on all SPs can be updated concurrently.

#### 7.2.10. Resistance to Double-Spending Attack

The SP reads the world state to obtain the latest transaction to ensure that mobile users can only use the latest access privileges. Each transaction records the registration and update time of user access privileges. Thus, the improper behavior of mobile users using expired privileges can be detected by the SP.

### 7.3. Security Features Analysis

This section evaluates the proposed scheme by comparing its security features with several related schemes [8,12,13,15]. Ref. [8] is the representative multi-server authentication scheme for MCC services. Ref. [15] is the latest RC-based multi-server authentication and authorization scheme for MCC hierarchical services. Ref. [12] is the newest blockchain-based multi-server authentication scheme for the mobile cloud environment. Ref. [13] is the latest distributed multi-authority ABE-based authentication and authorization scheme for cloud hierarchical services. Table 8 compares the security features among five schemes. The comparison results indicate that our scheme has better security attributes than the above related schemes.

## 8. Performance Analysis

### 8.1. Performance Analysis in the Authentication Phase

In this section, we compare the computation costs and communication efficiencies of our proposed scheme with the related multi-server authentication schemes for the MCC environment [8,12,15].

#### 8.1.1. Computation Comparison Analysis

This section compares the computation costs of our proposed scheme with other schemes [8,12,15] in the authentication phase, which is used most frequently. To obtain a credible result of the computation cost comparison, we continue to follow the running time of the computing operations mentioned in He et al.’s scheme and Xiong et al.’s scheme [8,15]. Using software implementation, the running time of one MAC operation and one fuzzy extraction are approximately equal to the running time of two hash operations and one scalar multiplication operation, respectively [12,50]. Table 9 indicates the notations, as well as their running time, of all of operations in our scheme and other related schemes.

(1) $T_{mp}$: The running time of one map-to-point hash operation;
(2) $T_{sm}$: The running time of one scalar multiplication operation in G;
(3) $T_{bp}$: The running time of one bilinear paring operation;
(4) $T_{pa}$: The running time of one point addition operation in G;
(5) $T_{exp}$: The running time of one exponentiation operation;
(6) $T_{h}$: The running time of an one-way hash operation;
(7) $T_{MAC}$: The running time of one MAC operation.
(8) $T_{fe}$: The running time of one fuzzy extraction operation.

**Figure 7:**
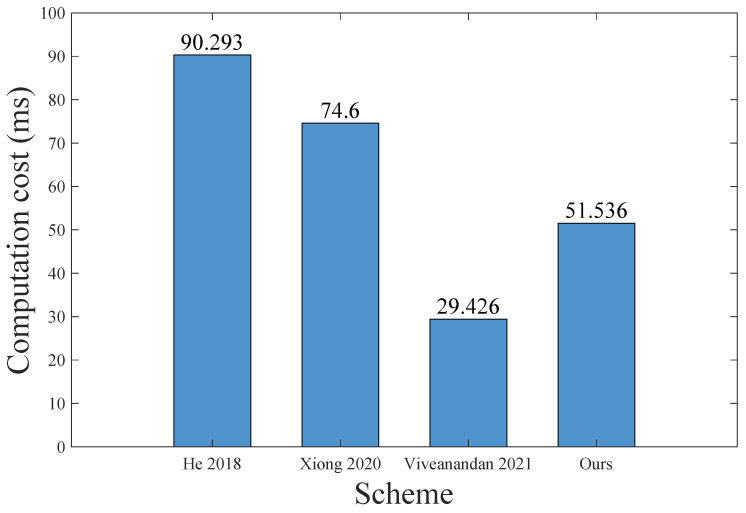
Computation cost versus scheme [8,12,15].

In Table 10, we calculated the computation cost of our scheme and the previous relevant schemes [8,12,15]. From the comparison in Table 8 and Table 10, and Figure 7, we conclude that, compared with the latest blockchain-based multi-server authentication scheme [12], our scheme is slower, but we use smart contracts to achieve the additional authorization function. In comparison to the representative multi-server authentication scheme [8] and the newest centralized multi-server authentication and authorization scheme [15], the computation cost in our scheme is reduced by 42% and 30%, respectively.

#### 8.1.2. Communication Comparison Analysis

This setcion evaluates the proposed scheme by comparing its communication cost with several related schemes [8,12,15]. We only compare the communication cost in the authentication phase used most frequently. Table 11 indicates the bit length of the data structure used in our scheme and other relevant schemes. In addition, we assume that the bit length of the access control level lv is 3, which is enough to represent eight kinds of access privileges.

**Figure 8:**
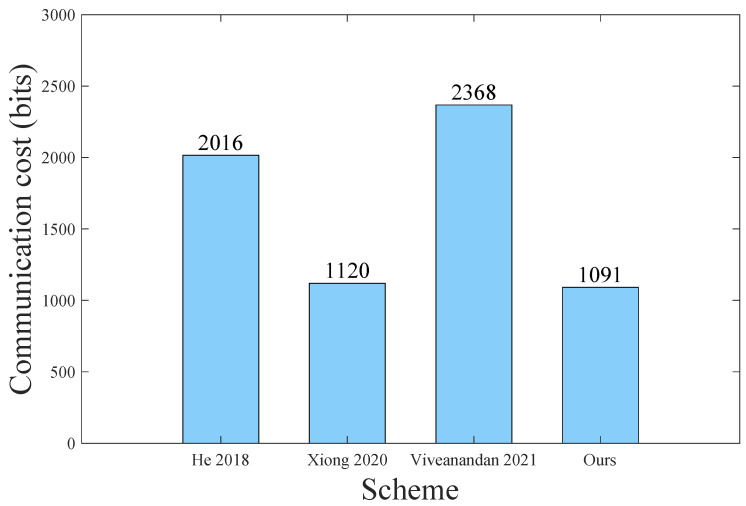
Communication cost versus scheme [8,12,15].

In the authentication phase of our scheme, the first message $\left\{X,M_{1},T_{3}\right\}$ requires 320 + (32 + 32 + 160 + 3) + 32 = 579 bits, and the second message $\left\{Y,M_{2},T_{4}\right\}$ requires 320 + 160 + 32 = 512 bits. The total communication cost of our scheme is 579 + 512 = 1091 bits. Table 12 illustrates the total communication cost of other related schemes in the authentication process. From the comparison in Table 12 and Figure 8, we find that our scheme is the most efficient in communication overhead. The reason for this is that our scheme requires minimal message exchange rounds.

### 8.2. Blockchain Storage Overhead Analysis

In this section, we analyze the impact of the number of transactions on the blockchain storage in our scheme and other related schemes. In other blockchain-based access control scenarios, a transaction is usually used to record one authorization relation between a subject and an object owner [21,22,26,27,28,29]. This type of transaction in this paper is called one to one. In our scheme, a transaction records multiple authorization relations between a mobile user (subject) and multiple SPs (object owners). This type in this paper is called one to many. The transaction structure $\left\{PID_{i},PK_{ui},ID_{sj}^{r},PK_{sj}^{r},L,T_{1},S_{ui},S_{sj}^{r}\right\}$ is a one to many type in our scheme. Reasonably, $\left\{PID_{i},PK_{ui},ID_{sj}^{r},PK_{sj}^{r},S_{n},P,Day,C,T_{1},S_{ui},S_{sj}^{r}\right\}$ is supposed to be a one to one type in our scheme.

#### 8.2.1. The Blockchain Storage Overhead in Our Scheme

In our blockchain structure, *m* mobile users are off chain and *n* SPs maintain the blockchain network. one to one type means that *n* transactions record a user’s access privileges on *n* SPs. one to many type means that one transaction record a user’s access privileges on *n* SPs. In the registration phase, the total number of transactions in the one to one type is $mn^{2}$. *m* users need mn transactions completing registration that are dispersedly stored on *n* SPs. The total number of transactions in the one to many type is mn. *m* users need *m* transactions completing registration that are dispersedly stored on *n* SPs. In the update phase, when each mobile user updates *k* times in total, the total number of transactions is shown in Table 13. Our scheme enables users to update the privileges on all SPs at once by using one transaction. However, if the one to one type updates the privileges on all SPs simultaneously, a user needs *n* transactions each time.

**Figure 9:**
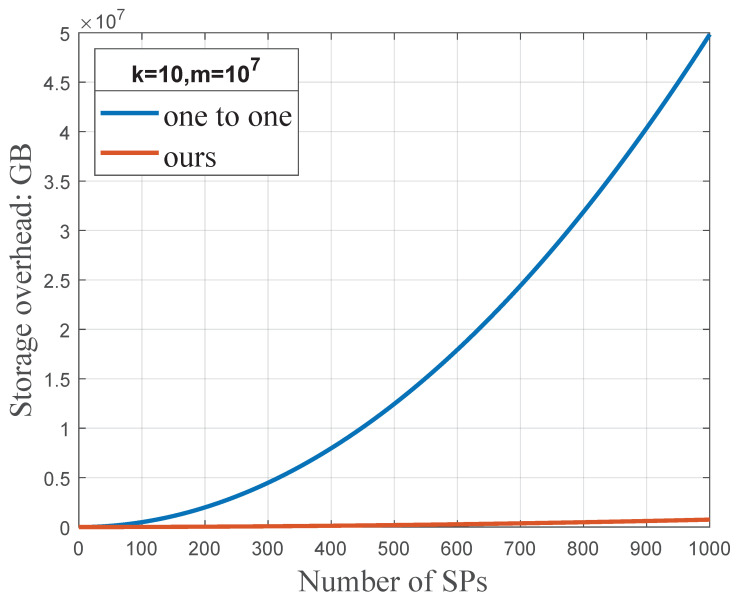
Storage overhead comparison of the increase in SPs.

**Figure 10:**
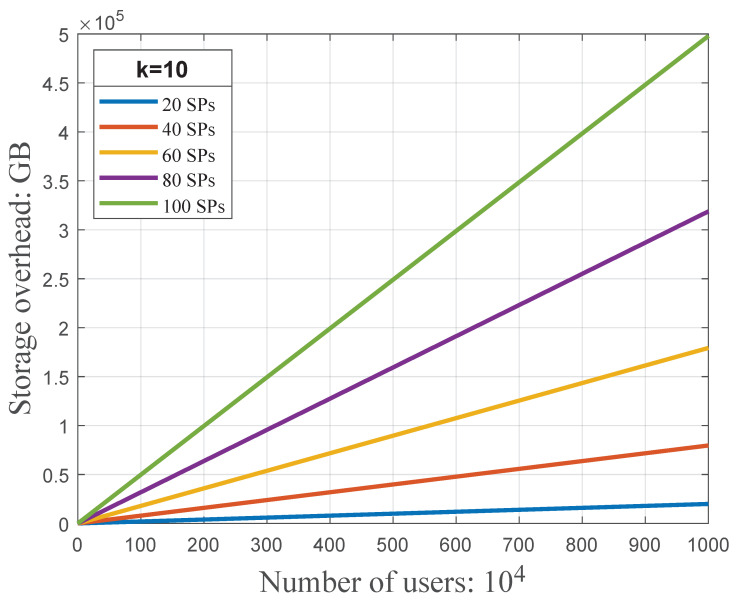
Storage overhead in onetoone type regarding the increase in users.

**Figure 11:**
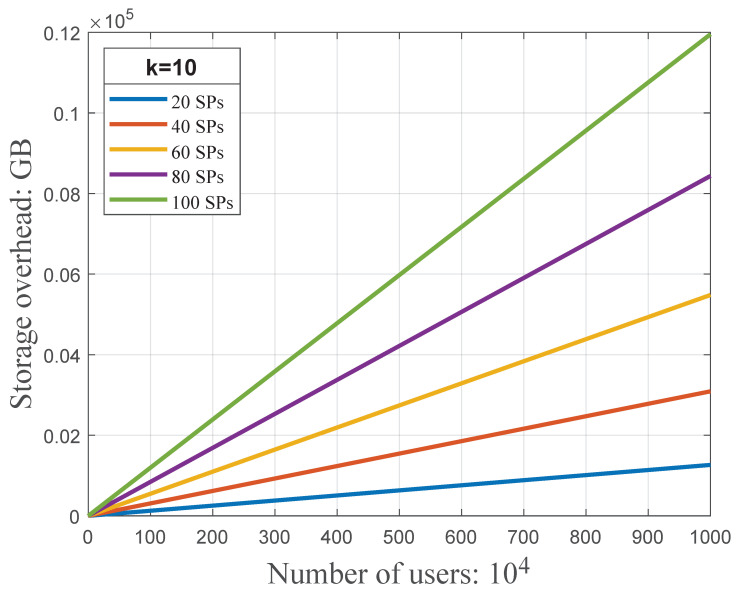
Storage overhead in ours regarding the increase in users.

We discuss the actual storage overhead of blockchain in both types. Suppose the bit length of the access privilege *p* is 3 bits, the service period Day is 10 bits, the cost *C* is 10 bits, and the signature is 320 bits. We test that one transaction of Fabric 2.3 in CouchDB is approximately 0.3KB. According to Table 11, the storage sizes of a transaction in one to one and one to many types are 3889 bits and 3834 + 55*n* bits separately. Thus, the total storage overhead of blockchain in one to one and one to many type is $\left(k+1\right)\;3889\;mn^{2}$ bits and $\left(k+1\right)\;\left(3834\;mn+55\;mn^{2}\right)$ bits, respectively. Figure 9 shows that, compared with the one to one type, the one to many type in our scheme requires less storage overhead and possesses a higher scalability when the number of SPs grows. Figure 10 and Figure 11 indicates the relationship between the total storage of blockchain and the growth in the number of users in the one to one type and ours under a different number of SPs. It can be seen that the blockchain storage overhead in ours is much less than in the one to one type.

#### 8.2.2. The Blockchain Storage Overhead in Related Schemes

Other blockchain-based access control schemes possess different blockchain structures and transaction structures. Hence, we conducted a qualitative analysis for the number of transactions to indicate the impact on blockchain storage. In addition, granting permission and accessing resources are two functions common to all schemes. Thus, we compared the number of transactions required for these two functions with other schemes. Assume that there are *m* subjects, *n* object owners, and *x* external nodes. We consider the situation where a subject wants to request access permissions from all object owners. In this way, there is the largest number of transactions in blockchain. Table 14 summarizes the number of transactions that grant permission in the one to one type and one to many type in three usual cases of blockchain structures.

- $Case_{1}$: *n* object owners constitute the blockchain network.
- $Case_{2}$: *x* external nodes constitute the blockchain network.
- $Case_{3}$: *m* subjects and *n* object owners constitute the blockchain network.

Table 15 shows the comparison results of the number of transactions with other schemes in the functions of granting permission and accessing resources [21,22,26,27,28,29]. It is worth noting that accessing resources is usually the most frequent operation. Therefore, if each resource access requires a transaction, it also puts a burden on the storage of the blockchain. Our scheme and some other schemes [27,28,29] avoid this insufficiency.

## 9. Discussion

In this section, we discuss the difference between the RC-based authentication and authorization scheme and our blockchain-based authentication and authorization scheme, single registration, blockchain storage, access privilege updates, and double-spending attack.

In the traditional RC-based multi-server authentication and authorization scheme, the server verifies the identity of the user and concurrently verifies whether the user has correct access privilege or not [9]. The integration of authentication and authorization at the same stage makes the cloud service system more efficient [15]. However, such a centralized scheme requires a trusted third party to issue identity and permission credentials for users, which makes it faces a single point of failure and have a high trust overhead. In our blockchain-based multi-server authentication and authorization scheme, the user can register on the arbitrary SP and obtain corresponding identity and permission credentials. It has the fault-tolerant capability to resist a single point of failure. In addition, our scheme uses the blockchain to establish the distributed trust relationship in the MCC environment, which avoids the use of RC, which needs to be completely trusted.

Single registration in the RC-based authentication and authorization scheme is straightforward. The user only needs to register on RC once and obtain one credential. All SPs trust the validity of credentials issued by RC for users and make authorization decisions according to the privileges in the credential. However, it is challenging to achieve single registration in our blockchain-based scheme without a trusted third party that distributes credentials for users. On the one hand, users need to prove the validity of the registration information submitted, e.g., public keys generated by themselves. On the other hand, SPs need to trust that a user registered on an arbitrary SP obtains the credential that can be used to access their services. Therefore, we used the NIZK proof of knowledge to force users to prove the discrete logarithm knowledge of the public key. Moreover, an automatic and transparent smart contract was applied to complete the single registration without the trusted third party.

Although the blockchain can enhance the security of the MCC system and reduce trust overhead, it has the issue of high storage overhead. Each node in the blockchain needs to store the same ledger, which leads to expensive storage overhead and restricts the system’s scalability. In particular, the total storage capacity is $n^{2}$ transactions, where the blockchain, which is maintained by *n* servers, needs to store a user’s *n* transactions that, respectively, record different access rights on *n* servers. Based on this storage feature, our scheme consolidates the user’s multiple access privileges into one transaction. In this way, the total storage capacity is *n* transactions since *n* servers, which maintain the blockchain, just need to store *n* transactions. In the MCC scenarios, the number of mobile users in the tens of millions emphasizes the importance of reducing the storage pressure on the blockchain.

The proposed scheme enables the mobile user to update access privileges anytime and anywhere. It brings convenience to users as well as the risks of double-spending attacks. In the access privilege update phase, the user submits new transactions that record new privileges to the blockchain. After the smart contract check, new transactions are written to the blockchain to complete the privileges update. However, old transactions that record old privileges cannot be deleted and modified due to the immutability of the blockchain. If no processing is made, the adversary can use the privileges in old transactions to access the service. In this paper, we used the world state to prevent this double-spending attack. The world state is stored in the key-value database and is the last transaction data about the key. In our solution, we used the user’s unique pseudonym “PID” as the key. In the authorization phase, the SP both uses the key “PID” to read the newest transaction content that records the newest privilege of the user and makes corresponding authorization decisions. In this way, the double-spending attack is prevented.

## 10. Conclusions

This paper proposes a blockchain-based authentication and authorization scheme for MCC hierarchical services. By using the zero-knowledge proof and the smart contract, we achieved user single registration without a trusted third party, which reduces trust overhead and resists the single point of failure. In addition, only one transaction is needed to load all authorization relationships between a mobile user and all SPs, which significantly reduces the number of transactions required to complete registration and updates. We implemented a flexible update strategy that provides convenience for users and avoids multiple updates. In addition, we used the world state to prevent the reuse of expired privileges in old transactions, i.e., the double-spending attack, and our scheme has more useful security features than several related schemes. The performance analysis indicates that our proposed scheme is efficient and scalable.

In our scheme, transactions are stored on the transparent blockchain in plaintext. The user’s registration information is publicly visible. Hence, our proposed scheme must work on the permissioned blockchain and cannot be directly used on the permissionless blockchain. In future work, we will focus on decentralized authorization with the storage of access privileges in the form of ciphertext, and we will introduce anonymous credential technology to enhance privacy.

## Figures and Tables

**Table 1:** Abbreviation and symbol.

Abbreviation/Symbol	Description
MCC	Mobile cloud computing
SP	Service provider
RC	Registration center
IoT	Internet of Things
ICN	Information-centric networking
NIZK	Non-interactive zero-knowledge
ABE	Attribute-based encryption
MSP	Membership service provider
$U_{i}$	Mobile user
$S_{j}$	Service provider
MD	Mobile device
$PID_{i}$	Pseudonym of $U_{i}$.
$ID_{sj}$	Identity of $S_{j}$.
$PW_{i}$	Password of $U_{i}$.
$SK_{ui}$, $SK_{sj}$	Private key of $U_{i}$, $S_{j}$.
$PK_{ui}$, $PK_{sj}$	Public key of $U_{i}$, $S_{j}$.
*L*	Service subscription list.
lv, lev	Access privilege of mobile user.
h(·)	One-way hash function
MAC(K,M)	Message authentication code
‖	Concatenation operation
⊕	Exclusive-OR operation

**Table 2:** Service subscription list *L*.

$L_{S}$	$L_{P}$	$L_{D}$	$L_{C}$
$S_{1}$	$Lev_{S1}$	$Day_{S1}$	$C_{S1}$
⋮	⋮	⋮	⋮
$S_{j}$	$Lev_{Sj}$	$Day_{Sj}$	$C_{Sj}$
⋮	⋮	⋮	⋮
$S_{n}$	$Lev_{Sn}$	$Day_{Sn}$	$C_{Sn}$

**Table 3:** User registration and subscription phase.

$U_{i}$ $S_{j}^{r}$
Generates $SK_{ui}$, *L*,
Calculates $PK_{ui}=SK_{ui}\cdot P$, $PID_{i}=h_{5}\left(PK_{ui}\right)$,
$S_{ui}=Sig(SK_{ui},PID_{i}\|T_{1}\left\|L\right)$
$\pi \leftarrow ZkPoK\{\left(SK_{ui}\right):PK_{ui}=$$SK_{ui}\cdot P$},
$\overset{\;\{PID_{i},PK_{ui},S_{ui},L,reg,T_{1},\pi \}\;}{\to }$
checks $|T_{2}-T_{1}|<\Delta T$ and π
If $Ver(PK_{ui},S_{ui},PID_{i}\|T_{1}\|L)=1$,
calculates $S_{sj}^{r}=Sig\left(SK_{sj}^{r},PID_{i}\right\|ID_{sj}^{r}$
$\|PK_{ui}\|PK_{sj}^{r}\left\|L\right\|T_{1})$
Then, $S_{j}$ submits the transaction
$\left\{PID_{i},PK_{ui},ID_{sj}^{r},PK_{sj}^{r},L,T_{1},S_{ui},S_{sj}^{r}\right\}$
to the blockchain.
Smart contract Algorithm 2 checks
whether $U_{i}$ has not registered,
and the correctness of *L*.
If it holds, one registration transaction
is written into the blockchain.
$\overset{\;registration\;success\;}{\leftarrow }$
Inputs and chooses $PW_{ui}$ and $b_{i}$,
computes $Z_{i}=h_{0}(PID_{i}\|PW_{ui}\left\|b_{i}\right)$,
$F_{i}=\left(SK_{ui}\|T_{1}\right)⊕Z_{i}$,
$V=h_{3}(h_{2}(SK_{ui}\|T_{1}\|Z_{i}))$
Stores $F_{i}$, *V*, $b_{i}$, $PK_{ui}$, π, and *L* into MD.

**Table 4:** Authentication and authorization phase.

$U_{i}$ $S_{j}$
Inputs $PID_{i}$, $PW_{ui}$, $b_{i}$
computes $Z_{i}=h_{0}(PID_{i}||PW_{ui}\left|\right|b_{i})$,
$SK_{ui}\|T_{1}=F_{i}⊕Z_{i}$,
$V_{0}=h_{3}(h_{2}(SK_{ui}\|T_{1}\|Z_{i}))$
If $V_{0}$=*V*, extracts $SK_{ui}$ and $PID_{i}$,
determines $lv\in L_{P}$ on $S_{j}$, according to list *L*.
Then, generates $\alpha \in Z_{q}^{*}$, computes X=α·P,
$H_{1}=h_{1}(X\|PID_{i}\|ID_{sj}\|T_{3}\left\|lv\right)$,
$st=\alpha +H_{1}SK_{ui}$mod*q*,
$M_{1}=(PID_{i}\|ID_{sj}\left\|st\|lv\right)⊕h_{4}\left(\alpha \cdot PK_{sj}\right)$,
$\overset{\;\{X,M_{1},T_{3}\}\;}{\to }$
checks $|T_{4}-T_{3}|<\Delta T$,calculates $PID_{i}\|$
$ID_{sj}\left\|st\right\|lv=M_{1}⊕h_{4}\left(SK_{sj}\cdot X\right)$.
$S_{j}$ uses the pseudonym $PID_{i}$ to extract
the transaction $\{PID_{i}^{*},PK_{ui}^{*},ID_{sj}^{r*},$
$PK_{sj}^{r*},L^{*},T_{1}^{*},S_{ui}^{*},S_{sj}^{r*}\}$ from key-value
database, verifies $st\cdot P\overset{?}{=}X+h_{1}(X$
$\|PID_{i}\|ID_{sj}\|T_{3}\left\|lv\right)\cdot PK_{ui}^{*}$.
If it holds, accepts $U_{i}$.
Then, checks lv and service period.
If $\left\lfloor T_{4}-T_{1}^{*}\right\rfloor <Day_{Sj}\in L_{D}^{*}$, $lev=Lev_{Sj}$.
If $\left\lfloor T_{4}-T_{1}^{*}\right\rfloor \geq Day_{Sj}\in L_{D}^{*}$, lev=0.
$S_{j}$ checks whether lev=lv. If it holds,
$S_{j}$ provides lev level of service.
Generates $\beta \in Z_{q}^{*}$, computes Y=β·P
$key=h_{3}(PID_{i}\|ID_{sj}\left\|X\|Y\|st\right\|$
β·X‖lev)
$M_{2}=MAC(key,PID_{i}\|ID_{sj}\left\|Y\right\|X$
$\|T_{4}\left\|lev\right)$
$\overset{\;\{Y,M_{2},T_{4}\}\;}{\leftarrow }$
Checks $|T_{5}-T_{4}|<\Delta T$, sets $lev^{\prime }=lv$, computes
$key^{\prime }=h_{3}(PID_{i}\|ID_{sj}\|X\|Y\|st\|\alpha \cdot Y\|lev^{\prime })$
$M_{2}^{\prime }=MAC(key^{\prime },PID_{i}\|ID_{sj}\left\|Y\|X\right\|T_{4}\left\|lev^{\prime }\right)$
Checks $M_{2}^{\prime }$=$M_{2}$. If it holds, accepts $S_{j}$.

**Table 5:** Platform development parameters.

Name	Parameters
CPU	I5-6300HQ 2.30 GHz
Memory	8.00 GB
Hard disk	1 TB
Operating system	Window 10
Programming tool	IntelliJ IDEA 2021.2
Virtual machine	VMware Workstation 16.0
Parameters (virtual machine)	Memory: 2 GB, Hard disk 30 GB, OS: Ubuntu 20.10
Blockchain	Hyperledger Fabric 2.3

**Table 6:** The list $L_{1}$ in the user registration and subscription phase.

$L_{S}$	$L_{P}$	$L_{D}$	$L_{C}$
$S_{1}$	1	1	1
$S_{2}$	1	2	8
$S_{3}$	3	3	9
$S_{4}$	0	0	0
$S_{5}$	0	0	0

**Table 7:** The list $L_{2}$ in the access privilege update.

$L_{S}$	$L_{P}$	$L_{D}$	$L_{C}$
$S_{1}$	0	0	0
$S_{2}$	2	1	1
$S_{3}$	3	3	3
$S_{4}$	0	0	0
$S_{5}$	0	0	0

**Table 8:** Security features comparisons.

Security Features	[8]	[15]	[12]	[13]	Ours
Single registration	✓	✓	✓	✓	✓
Mutual authentication	✓	✓	✓	✓	✓
User anonymity and un-traceability	✓	✓	✓	-	✓
Multi-factor security	✓	✓	✓	-	✓
Resistance to reply attack	✓	✓	✓	✓	✓
Resistance to wrong password login/update attack	✕	✓	✓	-	✓
Hierarchical access control	-	✓	-	✓	✓
Access within limits of permission	-	✓	-	✓	✓
Efficient and flexible update user access privilege	-	✓	-	-	✓
Resistance to double-spending attack	-	-	-	-	✓
Withstands single-point failure	✕	✕	✓	✓	✓

**Table 9:** Running time of operations (millisecond).

	The User	The SP
$T_{mp}$	33.582	5.493
$T_{sm}$	13.405	2.165
$T_{bp}$	32.713	5.427
$T_{pa}$	0.081	0.013
$T_{exp}$	2.249	0.339
$T_{h}$	0.056	0.007
$T_{MAC}$	0.112	0.014
$T_{fe}$	13.405	2.165

**Table 10:** Computation comparisons in authentication phase.

Scheme	Mobile User	SP	Total Cost
[8]	$T_{mp}+3T_{sm}+2T_{exp}+4T_{h}\approx 78.519$ ms	$2T_{bp}+2T_{pa}+2T_{exp}+5T_{h}\approx 11.774$ ms	90.293 ms
[15]	$T_{bp}+2T_{sm}+2T_{exp}+8T_{h}\approx 64.469$ ms	$T_{bp}+2T_{sm}+2T_{exp}+8T_{h}\approx 10.131$ ms	74.6 ms
[12]	$T_{pa}+T_{sm}+T_{fe}+6T_{h}\approx 27.227$ ms	$T_{sm}+T_{pa}+3T_{h}\approx 2.199$ ms	29.426 ms
Ours	$3T_{sm}+T_{MAC}+6T_{h}\approx 40.663$ ms	$5T_{sm}+T_{pa}+T_{MAC}+3T_{h}\approx 10.873$ ms	51.536 ms

**Table 11:** Bit length of data structure.

Notations	Length (Bits)
$PID_{i}$	32
$ID_{sj}$	32
Hash output	160
Random number	160
Timestamp T	32
ECC point	320
Digital signature	320

**Table 12:** Communication comparisons in authentication phase.

Scheme	Rounds of Exchange	Number of Bits
[8]	4	2016 bits
[15]	3	1120 bits
[12]	6	2368 bits
Ours	2	1091 bits

**Table 13:** Total transactions in our scheme.

Transaction Types	*One to One*	*One to Many*
Registration phase	$mn^{2}$	mn
Update phase	$mkn^{2}$	mkn
Total	$\left(k+1\right)mn^{2}$	(k+1)mn

**Table 14:** Total transactions in three cases.

Case Types	*One to One*	*One to Many*
$Case_{1}$	$mn^{2}$	mn
$Case_{2}$	mnx	mx
$Case_{3}$	mn(m+n)	m(m+n)

**Table 15:** Comparison results of the number of transactions.

Scheme	Scenario	Grant Permission	Access Resources
[21]	ICN	mnx	1
[22]	IoT	mn(m+n)	1
[26]	IoT	mnx	1
[27]	IoT	mn(m+n)	0
[28]	IoT	mn(m+n)	0
[29]	IoT	mn(m+n)	0
Ours	MCC	mn	0

## Data Availability

Not applicable.
